# The boundary for quantum advantage in Gaussian boson sampling

**DOI:** 10.1126/sciadv.abl9236

**Published:** 2022-01-26

**Authors:** Jacob F. F. Bulmer, Bryn A. Bell, Rachel S. Chadwick, Alex E. Jones, Diana Moise, Alessandro Rigazzi, Jan Thorbecke, Utz-Uwe Haus, Thomas Van Vaerenbergh, Raj B. Patel, Ian A. Walmsley, Anthony Laing

**Affiliations:** 1 Quantum Engineering Technology Labs, University of Bristol, Bristol, UK.; 2 Ultrafast Quantum Optics Group, Department of Physics, Imperial College London, London, UK.; 3 Quantum Engineering Centre for Doctoral Training, University of Bristol, Bristol, UK.; 4 Hewlett Packard Enterprise, Zurich, Switzerland.; 5 Hewlett Packard Enterprise, Amstelveen, Netherlands.; 6 HPE HPC/AI EMEA Research Lab, Wallisellen, Switzerland.; 7 Hewlett Packard Labs, HPE Belgium, Diegem, Belgium.; 8 Department of Physics, University of Oxford, Oxford, UK.

## Abstract

Identifying the boundary beyond which quantum machines provide a computational advantage over their classical counterparts is a crucial step in charting their usefulness. Gaussian boson sampling (GBS), in which photons are measured from a highly entangled Gaussian state, is a leading approach in pursuing quantum advantage. State-of-the-art GBS experiments that run in minutes would require 600 million years to simulate using the best preexisting classical algorithms. Here, we present faster classical GBS simulation methods, including speed and accuracy improvements to the calculation of loop hafnians. We test these on a ∼100,000-core supercomputer to emulate GBS experiments with up to 100 modes and up to 92 photons. This reduces the simulation time for state-of-the-art GBS experiments to several months, a nine–orders of magnitude improvement over previous estimates. Last, we introduce a distribution that is efficient to sample from classically and that passes a variety of GBS validation methods.

## Teaser

Methods are developed and benchmarked that substantially reduce the time to simulate cutting-edge quantum optics experiments.

## INTRODUCTION

A quantum advantage is typically considered to be achieved when a quantum experiment outperforms a classical computer at a computational task, with strong evidence of an exponential separation between quantum and classical run times. On the basis of plausible complexity conjectures, boson sampling (*1*, *2*) is a class of photonic experiments with potential to deliver quantum advantage. Measurement of correlated photon detection events constitutes sampling from a distribution with probabilities that correspond to classically intractable matrix functions. In Gaussian boson sampling (GBS) (*3*), squeezed states are injected into an interferometer, with subsequent photon detection producing correlation events that are related to matrix loop hafnians (*4*, *5*). A major advancement in experimental photonics was recently reported, in which a GBS experiment composed of 100 optical modes, named Jiŭzhāng (*6*), observed up to 76 photon detection events and claimed a quantum advantage. Once assembled, Jiŭzhāng ran in 200 s, while the best available classical algorithms running on the most powerful contemporary supercomputer would require 600 million years to simulate Jiŭzhāng.

While the theoretical proposal for GBS assumed the use of photon number–resolving detectors (PNRDs), experimental implementations frequently use threshold detectors, which click to distinguish between 0 and at least 1 photon. This does not affect the complexity of GBS provided that collisions (multiple photons arriving at the same detector) are unlikely (*7*). These events were assumed improbable and were neglected in the original proposal (*1*). Jiŭzhāng both uses threshold detectors and operates in a regime where there is a high probability of collisions between photons. The classical complexity for GBS with PNRDs in regimes with high collisions has recently been studied, albeit in a restricted setting (*8*). However, we are not aware of any attempts at benchmarking in this regime or any work covering investigating the complexity of collisions when measured using threshold detectors. This obfuscates the boundary for quantum advantage.

Here, we present classical algorithms that calculate exact, correlated photon detection probabilities for GBS simulations with PNRDs, in the presence of collisions, faster than existing methods. Furthermore, we introduce a new classical method to generate samples for GBS simulations with threshold detectors, which runs orders of magnitude faster than classical methods to generate samples with PNRDs, when collisions dominate. Both of these results provide quadratic speedups to prior methods in the high-collision regime. We apply these results to two sampling algorithms: a probability chain rule method (*9*) and Metropolis independence sampling (MIS) (*10*). We report nine–orders of magnitude reduction in the time taken to simulate idealized Jiŭzhāng-type GBS experiments with threshold detectors. This enabled us to classically simulate, on a ∼100,000-core supercomputer, GBS experiments with 100 modes and up to 60 click detection events. Replacing threshold detectors with PNRDs in this simulation allows us to generate a 92-photon sample but increases the run time significantly. This is a substantial improvement over previous attempts to simulate GBS (*11*–*13*), which reached around 20 photons. A review of GBS simulation methods can be found in the Supplementary Materials.

We find that simulating a 60-mode experiment with PNRDs is of comparable complexity to simulating a 100-mode experiment with the same density of photons and threshold detectors. Last, we develop and investigate a classically tractable distribution that passes a variety of canonical GBS verification tests, highlighting the importance of verifying GBS experiments against the most stringent adversarial tests available. By providing algorithms that exploit photon collisions to reduce the complexity of GBS and benchmarking these algorithms at large scales, we provide a reference point with which all experiments wishing to claim quantum advantage can be compared against. These results significantly sharpen the boundary of quantum advantage in GBS.

## RESULTS

### Loop hafnian algorithms

A particular detection event can be described by a photon number pattern $\vec{n}$, where *n_i_* is the number of photons in mode *i*. The probability of obtaining some $\vec{n}$ from a GBS experiment is

$$P(\vec{n})=\frac{P_{0}}{\prod_{i}n_{i}!}\text{lhaf}\;(A_{\vec{n}})$$ (1)

where *P*_0_ is the probability of measuring vacuum, lhaf ( · ) is the loop hafnian function, and $A_{\vec{n}}$ is a matrix that can be derived from $\vec{n}$ and the covariance matrix and displacement vector of the Gaussian state (see Materials and Methods). $A_{\vec{n}}$ is a 2*N* × 2*N* matrix, where *N* = ∑*_i_n_i_*. However, for a pure Gaussian state, $A_{\vec{n}}$ is block diagonal, with blocks $B_{\vec{n}}$ and $B_{\vec{n}}^{*}$, in which case

$$\text{lhaf}\;(A_{\vec{n}})={|\text{lhaf}\;(B_{\vec{n}})|}^{2}$$ (2)

$B_{\vec{n}}$ is an *N* × *N* matrix, so it is considerably faster to calculate its loop hafnian compared to $A_{\vec{n}}$. While a realistic GBS experiment will not produce a pure state, a Gaussian mixed state can be expressed as a statistical ensemble of pure states with differing displacement vectors (*9*, *14*); so, for the purposes of a sampling algorithm, it is generally possible to randomly choose a complex displacement vector $\vec{\alpha }$ from the correct distribution and then sample from the corresponding pure state. Hence, the computational complexity of generating a sample is set by the calculation of an *N* × *N* loop hafnian, $\text{lhaf}\;(B_{\vec{n}})$.

**Fig. 1.**
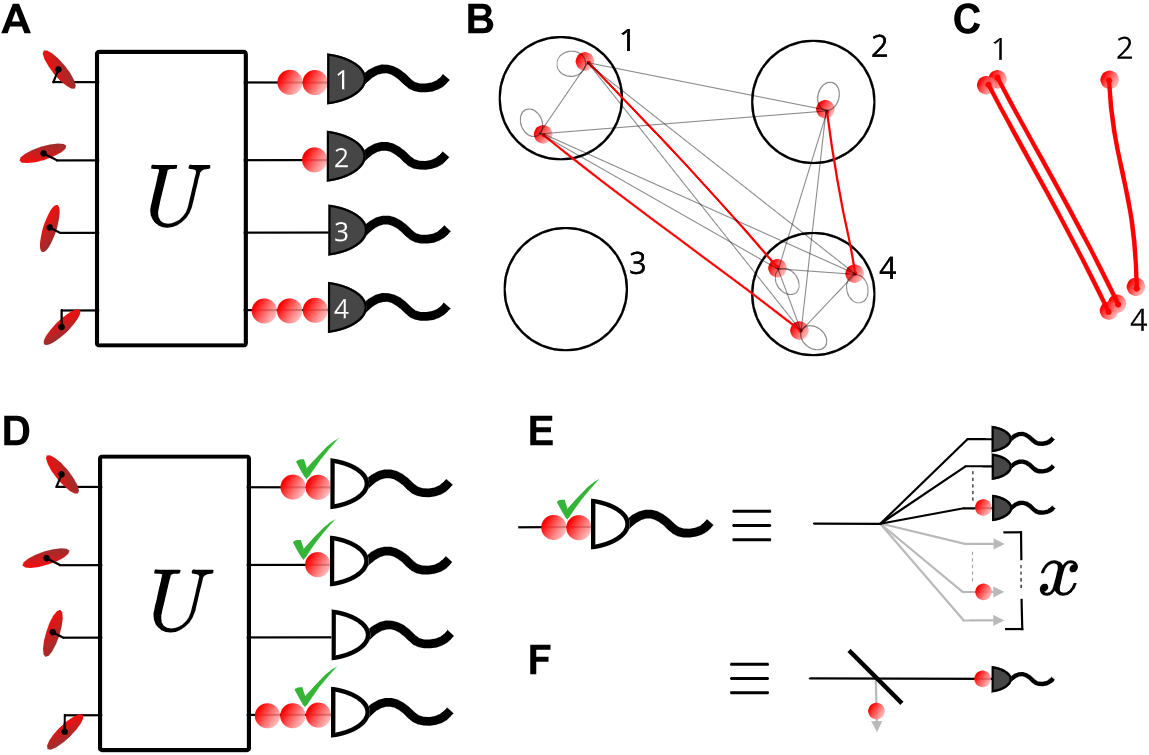
Conceptual schematic of repeated-row loop hafnian algorithm and threshold detector sampling method. (**A**) A GBS outcome with collisions, measured with PNRDs. (**B**) To calculate the associated probability, we group the photons into pairs (red lines) to maximize the number of repeated, identical pairs. (**C**) An inclusion/exclusion formula, or a finite-difference sieve, can then operate on the resulting pairs, with repeated pairs leading to a speedup. (**D**) The same event measured with threshold detectors, with “clicks” shown as green ticks. (**E**) We consider a fan-out to an array of subdetectors, with none likely to receive >1 photon. We can ignore the outcomes of all but the first detector to see a photon. *x* is introduced as the relative position of the detected photon and is also the fraction of the subdetectors that are ignored. (**F**) The probability of detecting the first photon at position *x* can be expressed as a loss followed by single-photon detection.

The fastest known algorithms for the loop hafnian run in exponential time using an inclusion/exclusion formula similar to the Ryser algorithm for the permanent (*15*). In boson sampling with Fock state inputs, Ryser can be generalized to take advantage of collisions, reducing the number of inclusion/exclusion terms to calculate from 2*^N^* to ∏*_i_*(*n_i_* + 1) (*16*–*18*). The repeated-moment formula for the loop hafnian achieves the same scaling for GBS (*19*). However, there is a much faster formula for general loop hafnians: The eigenvalue trace algorithm performs inclusion/exclusion on pairs of photons and so requires only 2^*N*/2^ terms (*20*, *21*). Here, we generalize eigenvalue trace to take advantage of collisions, reducing the number of terms to ∏*_i_*(η*_i_* + 1), where η*_i_* is the number of times a particular pairing of photons is repeated. This is lower-bounded by $\prod_{i}\sqrt{n_{i}+1}$ and upper-bounded by 2^*N*/2^. The grouping of photons into pairs is arbitrary, so we make use of a greedy algorithm to choose repeated pairings, reducing the number of inclusion/exclusions steps to as close to the lower bound as possible (see the Supplementary Materials). Figure 1 (A to C) shows how, when collisions occur in more than one mode, repeated pairs can be formed. In this example, $\vec{n}=(2,1,0,3)$, which is arranged into two pairings, one of which is repeated. This gives a sum over (2 + 1)(1 + 1) = 6 terms, reduced from 8 using eigenvalue trace (*20*), and compared to 24 using the repeated-moment algorithm (*19*).

We also present a loop hafnian formula that uses a finite-difference sieve instead of an inclusion/exclusion formula, like the Glynn formula for the permanent (*22*–*24*). This significantly improves the numerical accuracy with only a minor time penalty. Whereas accuracy is an issue for eigenvalue trace when *N* > 50 (*9*), the finite-difference sieve method has relative error <10^−8^ when tested up to *N* = 60 (see the Supplementary Materials). This allows us to maintain accuracy for large loop hafnians while using a conventional 128-bit complex floating point data type, which is desirable for speed and portability. We therefore use the finite-difference sieve formula for all benchmarking results presented in the “Benchmarking” section.

### Threshold detectors

When threshold detectors are used, the detection probabilities can be calculated using the Torontonian matrix function (*7*), which involves a sum over 2^*N*_c_^ terms, where *N*_c_ is the number of clicks (outputs with one or more photons). However, calculating this quantity is not necessarily the fastest approach to sampling threshold detection patterns. For a sufficiently low density of photons, it may be faster to simulate PNRDs and then simply reduce each nonzero photon number to a click. We show that it is possible to improve this, for any density of photons, to the level of an *N*_c_ × *N*_c_ loop hafnian, containing 2^*N*_c_^^/2^ terms.

We consider the detection system depicted in Fig. 1E. The mode is uniformly fanned out to many PNRD subdetectors such that the probability of a collision in any one subdetector can be neglected. This system provides a conceptual bridge between threshold detection and number-resolved detection (*25*). If these subdetectors within a mode are sampled sequentially, then once a single photon is seen, that mode registers a click. The remaining subdetectors, which have not yet been sampled, can be ignored because no more information is required about that mode. Hence, the number of detected single photons to simulate is *N*_c_, which sets the size of loop hafnian calculation. *x* is introduced as an additional variable giving the position of the single photon within the fan-out, normalized to vary between 0 and 1. As a result, a fraction *x* of the subdetectors are ignored; this can be related to applying a loss of *x* to the mode before detecting a single photon, as shown in Fig. 1F.

### Sampling algorithms

#### Chain rule sampling

These methods can be applied directly to the chain rule for simulating GBS described in (*9*) and in Materials and Methods. Here, the photon number in each mode is sampled sequentially, conditioned on the photon numbers in the previous modes. Finding the conditional probability distribution for mode *j* requires calculating the joint probabilities of (*n*_1_, …, *n_j_*) for all values of *n_j_* up to *n*_cut_, where *n*_cut_ is some cutoff such that the probability of having a greater number of photons can be neglected. Because *n*_cut_ should generally be several times larger than the expected number of photons, the speedup for calculating collision probabilities is especially applicable here. Furthermore, we make use of a batched method for simultaneously calculating all of the loop hafnians required for different values of *n_j_*, with approximately the same run time as calculating the largest loop hafnian, where *n_j_* = *n*_cut_ (see the Supplementary Materials). When simulating threshold detectors, we choose to reduce *n*_cut_ for each subdetector to 1. We again use a batching method to more efficiently sample different subdetectors within the same mode, which largely offsets the additional overhead from sampling several subdetectors per mode.

#### Metropolis independence sampling

We also investigate MIS, a Markov chain Monte Carlo method, for generating GBS samples. Here, samples *s_i_* are drawn from a proposal distribution, where *s_i_* is the *i*th sample in the chain. They are then accepted with probability

$$p_{\text{accept}}=\text{min}(1,\frac{P(s_{i})Q(s_{i-1})}{P(s_{i-1})Q(s_{i})})$$ (3)

where *P*(*s_i_*) is the target probability distribution, in this case that of ideal GBS, while *Q*(*s_i_*) is the proposal probability distribution, i.e., the probability of proposing a particular *s_i_*. If a proposed sample is rejected, then the previous sample is repeated, *s_i_* = *s*_*i* − 1_. This update rule ensures that the chain will converge toward the target distribution, which is its equilibrium state (*26*, *27*). Usually, some burn-in time, τ_burn_, is used to allow the chain to converge. As sequential samples are not independent, some thinning interval, τ_thin_, can also be used to suppress the probability of seeing repeated samples, keeping only one in every τ_thin_ sample. These parameters are critical to the efficiency of MIS and can generally be improved by choosing a proposal distribution that is close to the target distribution.

We expand our sample space so that *s_i_* contains the photon number pattern $\vec{n}$ and the complex displacement vector $\vec{\alpha }$. For the proposal samples, we draw $\vec{\alpha }$ from the correct distribution for the desired mixed state and then generate $\vec{n}$ from an “independent pairs and singles” (IPS) distribution based on the resulting pure state (see the Supplementary Materials). This distribution, which we introduce in this work, can be sampled from efficiently and has probabilities given by *N* × *N* loop hafnians of positive matrices. As an aside, we observe that the IPS distribution is already sufficient to pass many GBS verification methods (see the Supplementary Materials). The run time per sample is dominated by the two loop hafnians in *P*(*s_i_*) and *Q*(*s_i_*), with *P*(*s*_*i* − 1_) and *Q*(*s*_*i* − 1_) already calculated in the previous step.

When simulating threshold detectors with MIS, we take the continuum limit of a large number of subdetectors and introduce *x* as an additional continuous random variable that gives the position of the “first” detected photon within each mode with nonzero photons. Given a proposed photon number pattern, $\vec{x}$ can be sampled efficiently from its conditional distribution $p(\vec{x}|\vec{n})$. *P*(*s_i_*) and *Q*(*s_i_*) are then calculated with *N*_c_ × *N*_c_ loop hafnians, tracing out the unused subdetectors. One subtlety is that tracing out reintroduces mixture into the quantum state, so it is necessary to sample a further adjustment to the displacement vector $d\vec{\alpha }$ to obtain a pure state. This is only used in the calculation of *P*(*s_i_*). Details are given in the Supplementary Materials.

While we have chosen here to investigate MIS, other Monte Carlo methods may also be relevant such as rejection sampling (*28*). These methods may improve the run time, but we do not believe that they would change the asymptotic complexity.

### Benchmarking

**Fig. 2.**
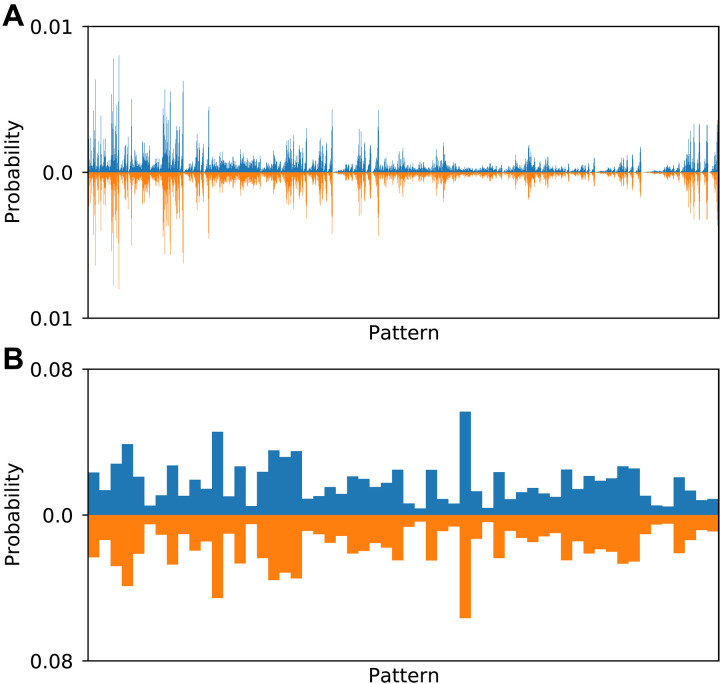
Theoretical and sample-estimated GBS probability distributions. Probability distribution for all six-photon detection outcomes for an eight-mode PNRD GBS simulation (**A**) and an eight-mode, three-click threshold detector GBS simulation (**B**). Blue bars show estimated probabilities using MIS; orange bars show exact probabilities.

To benchmark these methods, we choose parameters similar to those of Zhong *et al.* (*6*), while varying the system size by choosing the number of modes *M*. For the interferometer transformation, we sample a Haar random unitary matrix (*29*). The interferometer is fed with *M*/4 sources of two-mode squeezed vacuum, for which we choose a uniform squeezing parameter *r* = 1.55 and overall transmission η = 0.3 (70% loss). To demonstrate the correctness of our methods, we first test them on an *M* = 8 example, which is small enough that the results can be compared to the exactly calculated distributions. Figure 2 shows the accumulated distribution from 10^6^ samples with total photon number *N* = 6, generated by MIS for PNRD, along with the exactly calculated distribution. The total variation distance $\text{TVD}(p,q)=\frac{1}{2}\sum_{i}|p_{i}-q_{i}|=0.0153$, which is consistent with statistical uncertainties. With threshold detectors, we find that the TVD for the *N*_c_ = 3 distribution is 2.9 × 10^−3^, which benefits from the smaller statistical uncertainty due to the smaller number of possible outcomes. For the chain rule algorithm, we produce 10^6^ samples with both PNRD and threshold detectors. For PNRD with a cutoff of 12 photons, there were 74,973 samples with *N* = 6 from 10^6^ total samples, giving a TVD = 0.0554. For threshold detectors with 12 subdetectors, there were 195,150 *N*_c_ = 3 samples, and these gave a TVD = 0.0138. The larger TVDs are explained by the smaller sample size of the postselected distributions.

**Fig. 3.**
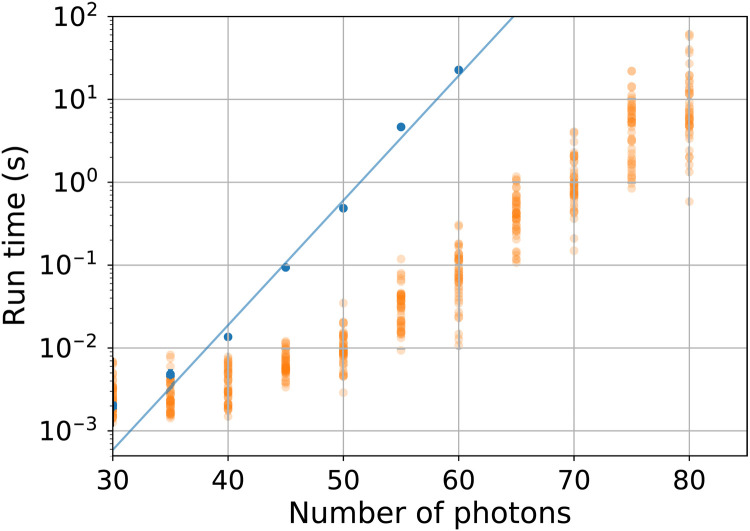
Loop hafnian run time benchmarking. Run time using the HPE benchmarking system, comparing eigenvalue trace loop hafnian algorithm on *N* × *N* matrices with and without speedup due to collisions (orange and blue dots). The blue line is an exponential fitted to the blue points. Collisions are determined by generating 39 samples for each *N* from the IPS distribution on 60 modes.

For large-scale tests, we make use of an internal HPE Cray EX benchmarking system consisting of 1024 nodes. A typical node is equipped with two AMD EPYC 7742 64-core processors clocked at 2.25GHz, and the nodes are interconnected with the Cray Slingshot 10 high-performance network. We first benchmark our loop hafnian formula on proposed IPS samples for an *M* = 60 example. The run time as a function of *N* is shown in Fig. 3, along with timings for the basic formula without speedup due to collisions. Making use of collisions generally improves the run time by one to two orders of magnitude for this range and allows 80-photon probabilities to be calculated in comparable time to a 60-photon probability without collisions. However, there is a large variation in run time between samples with the same *N*, depending on the amount of collisions in any particular configuration of the sampled photons. On the other hand, the run time for a loop hafnian without speedup from collisions shows little variation from 𝒪(*N*^3^2^*N*/2^) scaling, at least for *N* > 40.

**Fig. 4.**
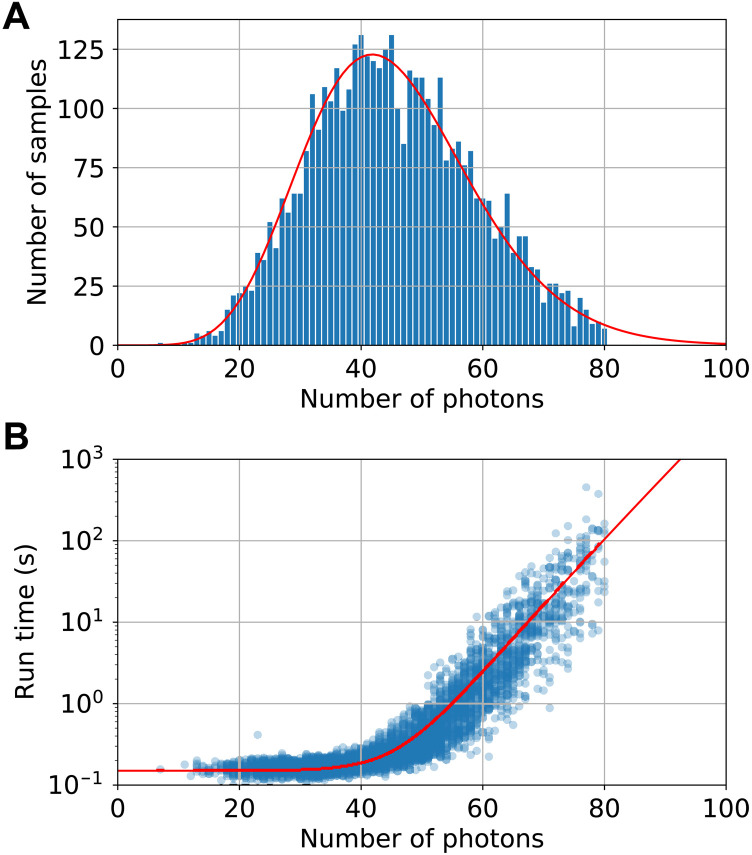
Chain rule benchmarking on 60-mode PNRD GBS. Chain rule simulation of *M* = 60 experiment with PNRDs. (**A**) Number of samples as a function of photon number, with the theoretically calculated distribution (red line) and (**B**) run time versus number of photons fitted with an exponential plus a constant (red line).

Using chain rule sampling, we simulate an *M* = 60 experiment with PNRDs, setting *n*_cut_ = 12 and an additional global cutoff of 80 photons. We generate 4200 samples in ∼3 hours. The global cutoff has no effect on the probability distribution of samples below the cutoff and is used to keep the run time per sample constrained. Figure 4A shows a histogram of the number of samples against number photons, which is in good agreement with the calculated distribution. Figure 4B shows the corresponding run times of the samples. Below ∼45 photons, the sample time appears approximately constant, suggesting that the problem size is not large enough to take full advantage of the system. Beyond that, the run time increases rapidly, although there is a wide range of variation depending on the particular configuration of output photons. We provide a rough fit line to this scaling, equal to (0.15 + 1.59 × 10^−9^ × *N*^3^*e*^0.147*N*^)s. Using this to extrapolate to photon numbers >80, we estimate that the average time per sample is ∼10 s. With the ∼66 times larger number of central processing units available in Fugaku, the world’s top-ranked supercomputer, this could be reduced to 130 ms.

**Fig. 5.**
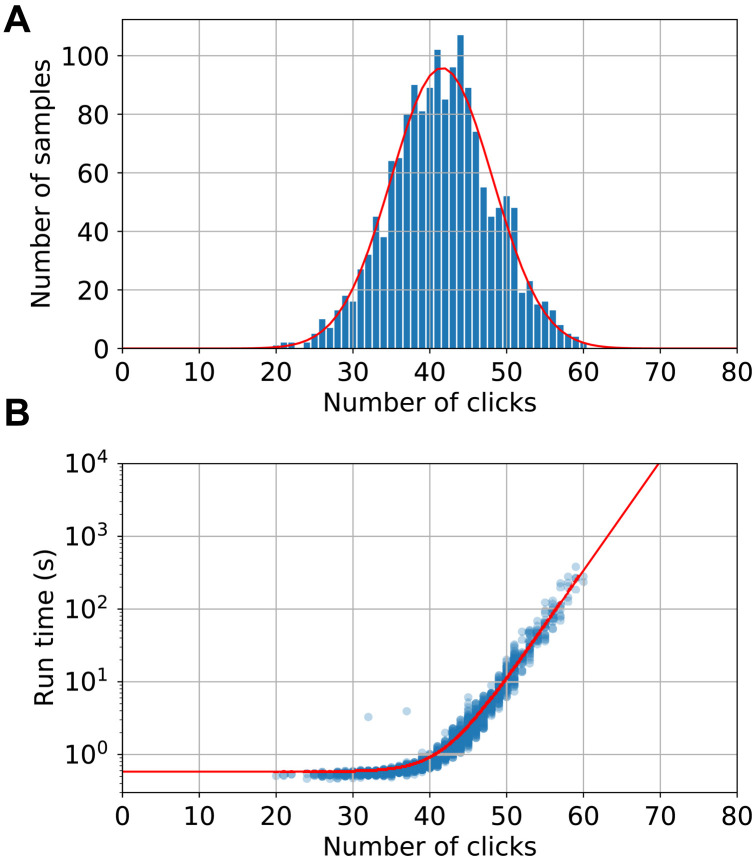
Chain rule benchmarking on 100-mode threshold detector GBS. Chain rule simulation of *M* = 100 experiment with threshold detectors. (**A**) Number of samples as a function of number of clicks, fitted with a Gaussian (red line). (**B**) Run time versus number of clicks, fitted with an exponential plus a constant (red line).

We then test chain rule simulation of an *M* = 100 experiment with threshold detectors, using 12 subdetectors per mode, and a global cutoff of 60 clicks. We generate 1600 samples in ∼3.5 hours. Figure 5A shows the histogram of click numbers, and Fig. 5B shows the corresponding run times of the samples. Beyond ∼45 clicks, the sample time increases approximately exponentially, from which we extrapolate to click numbers >60. The run times are fitted with a line (0.58 + 3.15 × 10^−7^ × 2^*N*/2^)s. From this, we predict that the mean time per sample is 8.4 s. On Fugaku, this could be reduced to around 127 ms.

On the basis of the scaling of the loop hafnian calculation and the distribution of samples over number of clicks, the estimated average time per MIS step is 0.45 s for an *M* = 100 system with threshold detectors. On Fugaku, this could be reduced to 7 ms, which is somewhat faster than generating a sample through the chain rule. However, the raw MIS chain will contain a high frequency of repeated samples due to rejections of the proposal sample; for some applications, this may be unimportant, but it would provide a clear difference from a true GBS experiment, where repeated samples are highly unlikely. In the Supplementary Materials, we investigate the τ_thin_ required to suppress repeated samples and find that it increases rapidly with system size, such that for *M* = 100, it is likely to be in excess of 600. Hence, if independently distributed samples are required, then the chain rule method is most likely preferable.

## DISCUSSION

Our results provide a new reference point for classical run times of GBS, an improved understanding of the classical complexity, and could improve verification techniques by making it practical to generate small numbers of samples from the distribution of much larger-scale experiments. Our IPS proposal distribution generates samples in polynomial time and is a better approximation than the standard adversarial models in the verification of GBS. As reported in the Supplementary Materials, the IPS distribution is largely able to pass the quantitative tests of GBS used in (*6*), which suggests a need for stronger verification methods, at the least, using IPS as a classically simulable adversary.

For GBS with threshold detectors, we have shown that the complexity can be reduced quadratically from $O(N_{c}^{3}2^{N_{c}})$ to $O(N_{c}^{3}2^{N_{c}/2})$. Comparing to the experiment of Zhong *et al.* (*6*), where 50 million samples were accumulated in 200 s, our 100-mode chain rule simulation implies that the classical run time can be reduced to ∼73 days. With announced plans for exascale computing on the horizon, for example, the planned system Frontier at the Oak Ridge National Laboratory, this could be further reduced to around 3 weeks. This does not diminish the experimental achievement of large-scale GBS from Zhong *et al.* (*6*), which remains faster than classical methods on supercomputers, if the time required for circuit programming (or, in the present case, fabricating a new, fixed interferometer) is not included. However, it has previously been reported that in boson sampling with Fock state inputs, at least 50 photon events are required to extend beyond the reach of an exact classical simulation in a reasonable time scale (*10*); for collision-free GBS, this threshold has been reported as being around 100 photons (*9*). We have now demonstrated that for GBS with threshold detectors, the number of correlated detector clicks should also be around 100. For GBS with PNRDs, the number of photons required to surpass this classical threshold will depend on the amount of collisions but must be ≥100.

Future claims to quantum advantage in GBS experiments might include increasing the level of programmability (*30*) and including PNRDs, which our results suggest adds significantly to the complexity, thus providing an alternative route to a larger quantum advantage than increasing the size of threshold detector experiments. For example, our 60-mode PNRD chain rule simulation ran in comparable time to the 100-mode threshold detector simulation. Meanwhile, a 100-mode PNRD simulation proved impractically slow even on the HPE Cray EX benchmarking system; we generated a single 92-photon event in 82 min.

Our algorithms are able to simulate the ideal operation of an experiment; they are near exact, down to Fock-basis truncation and finite precision errors. Any real experiment will feature noise and imperfections such as loss and nonideal interference. We already include a realistic level of loss in our benchmarking, and nonideal interference could be included if needed. Recent work has shown that efficient classical algorithms that approximate GBS can outperform the experiment because of its relatively high level of noise (*31*). However, it is important to note that these efficient algorithms cannot continue to outperform the experiment if sufficient reduction to experimental imperfections can be achieved. Furthermore, some classical simulation methods for boson sampling actively exploit errors such as loss and imperfect distinguishability to improve their run time (*32*–*36*). In future work, it may be possible to combine our methods with approximations that exploit errors to allow a faster simulation of an imperfect experiment.

## MATERIALS AND METHODS

### Gaussian boson sampling

This section outlines the methods for calculating photon number statistics in GBS. A derivation for the zero-displacement version of these formulae is given in (*4*). For displaced Gaussian states, we use the methods in (*5*, *37*). The Wigner function of an *M*-mode Gaussian state can be efficiently represented by using the 2*M* length mean vector **R**, and the 2*M* × 2*M* covariance matrix ***V***, of the canonical position and momentum operators $\vec{q}$ and $\vec{p}$. Equivalently, it can be represented in terms of creation and annihilation operators $a=(\begin{array}{c} \vec{a}\\ {\vec{a}}^{†} \end{array})$ as a complex valued displacement **α** and covariance matrix **σ** (*38*)

$$\alpha_{i}=\langle a_{i}\rangle$$ (4)

$$\sigma_{i,j}=\frac{1}{2}(\langle a_{i}a_{j}^{†}\rangle +\langle a_{j}^{†}a_{i}\rangle )-\alpha_{i}\alpha_{j}^{*}$$ (5)

Transformations between these bases are linear, as we can write $a=(q+\mathrm{ip})/\sqrt{2\hbar }$ and $a^{†}=(q-\mathrm{ip})/\sqrt{2\hbar }$. For details on how to construct these covariance matrices, we recommend reading (*4*, *14*, *25*) and the tutorial by Olivares on “Quantum optics in the phase space” (*39*).

We further define **σ***_Q_* = **σ** + **I**/2 as the complex-valued covariance matrix of the state’s Husimi *Q* function, $O=(I-\sigma_{Q}^{-1})$

$$X=(\begin{array}{cc} 0 & I\\ I & 0 \end{array})$$ (6)

***A*** = ***XO***, and $\gamma =\alpha^{†}\sigma_{Q}^{-1}$.

Probabilities of measuring photon number patterns $\vec{n}$ with PNRDs are now given by

$$P(\vec{n}|\sigma ,\alpha )=\frac{\text{exp}\;(-\frac{1}{2}\alpha^{†}\sigma_{Q}^{-1}\alpha )}{\sqrt{\text{det}\;(\sigma_{Q})}\prod_{i}n_{i}!}\text{lhaf}\;(A_{\vec{n}})$$ (7)

where lhaf ( · ) is the loop hafnian function. $A_{\vec{n}}$ is formed from ***A*** by repeating the *i*th and (*i* + *M*)th rows and columns *n_i_* times, and similarly, the *i*th and (*i* + *M*)th entry in **γ** is repeated *n_i_* times to form $\gamma_{\vec{n}}$. Then, the diagonal elements of $A_{\vec{n}}$ are replaced by the elements of $\gamma_{\vec{n}}$ because the weights of the loops are given on the diagonal of the matrix.

For pure states, $A_{\vec{n}}$ can be written in block form as

$$A_{\vec{n}}=(\begin{array}{cc} B_{\vec{n}} & 0\\ 0 & B_{\vec{n}}^{*} \end{array})$$ (8)

Here, $B_{\vec{n}}$ is a symmetric *N* × *N* matrix, with *N* the total photon number. As a result,

$$\text{lhaf}\;(A_{\vec{n}})={|\text{lhaf}\;(B_{\vec{n}})|}^{2}$$ (9)

so probabilities from a pure state can be calculated using loop hafnians of matrices of half the size compared to a mixed state.

### Sampling pure Gaussian states from mixed Gaussian states

Using the Williamson decomposition, we can write the covariance matrix as, ***V*** = ***SDS****^T^*. Here, ***D*** is a diagonal covariance matrix describing a thermal state in each mode, and ***S*** defines a symplectic transformation. Hence, any mixed Gaussian state can be written as a pure channel acting on thermal states.

By defining $T=\frac{\hbar }{2}SS^{T}$, a covariance matrix of a pure Gaussian state, and $W=S(D-\frac{\hbar }{2}I)S^{T}$, a covariance matrix describing the Gaussian classical noise added to the state, we can now write the original covariance matrix as ***V*** = ***T*** + ***W*** (*9*, *14*).

For the purposes of sampling the state, we can choose a pure state with vector of means ***R***^′^ sampled from the multivariate normal distribution described by covariance matrix ***W*** and means ***R***. This results in a pure state with covariance matrix given by ***T*** and means given by ***R***^′^. To sample from the multivariate normal distribution, we use the Cholesky decomposition of the covariance matrix (*40*).

### Chain rule GBS sampler

In this section, we describe our chain rule sampling algorithm. Probability chain rule methods were first shown to be useful for simulating boson sampling by Clifford and Clifford (*41*) and then later applied to GBS (*11*). We build upon the methods introduced in (*9*).

Sampling using the chain rule for probability proceeds by choosing part of the sample (in this case, e.g., the number of photons in the first mode) from its marginal probability distribution, then fixing this, and choosing the next part (e.g., number of photons in the second mode) from its conditional probability distribution depending on the first part. This is expressed as

$$P(n_{1},n_{2})=P(n_{1})P(n_{2}|n_{1})$$ (10)

This allows samples to be built up from distributions with very large numbers of possible outcomes, without calculating the probability of every possible outcome. In GBS, a difficulty is that the marginal probabilities are equivalent to probabilities from a mixed quantum state, and these are quadratically harder to calculate than for a pure state. This is because $A_{\vec{n}}$ is not block diagonal for mixed states, so its loop hafnian must be calculated directly.

To circumvent this, we simulate a measurement on all the modes in the coherent state basis. From this, we obtain a set of coherent state amplitudes $\vec{\beta }$ as measurement outcomes. We are free to ignore any given mode’s measurement outcome and choose to instead simulate its measurement in the photon number basis, conditional on the other mode’s measurement outcomes. Using this, we progressively replace each element of $\vec{\beta }$ by photon numbers $\vec{n}$. Sampling in the coherent state basis does not add to our complexity, as this is a Gaussian operation and so can be efficiently simulated using the Gaussian state formalism. Computing the conditional Gaussian states involves calculating the Schur complement of the state’s covariance matrix (*14*).

The procedure is as follows (*9*):

1) Sample modes 2 to *M* in the coherent state basis, obtaining a sample from the distribution *P*(β_2_, …, β*_M_*).

2) Sample the photon number in the first mode from the distribution *P*(*n*_1_∣β_2_, …, β*_M_*).

3) For *m* = 2 to *M* – 1, (i) Begin with a sample from the intermediate distribution: *P*(*n*_1_, …, *n*_*m*−1_, β*_m_*, …, β*_M_*). (ii) Discard the coherent state amplitude β*_m_*, and replace it with a photon number *n_m_* drawn from the distribution *P*(*n_j_*∣*n*_1_, …, *n*_*m*−1_, β_*m*+1_, …, β*_M_*). (iii) This leaves a sample drawn from the distribution *P*(*n*_1_, …, *n_m_*, β_*m*+1_, …, β*_M_*) that can be used as a starting point for the next step.

4) Discard β*_M_*, and replace it with *n_M_*, drawn from *P*(*n_M_*∣*n*_1_, . . , *n*_*M*−1_). This leaves a photon number sample drawn from the distribution *P*(*n*_1_, …, *n_M_*).

A proof showing that this algorithm samples from the GBS distribution is given in (*9*). To sample from *P*(*n_m_*∣*n*_1_, …, *n*_*m*−1_, β_*m*+1_, …, β*_M_*), the joint probabilities *P*(*n*_1_, …, *n_m_*, β_*m*+1_, …, β*_M_*) are calculated for all *n_m_* between zero and some finite cutoff *n*_cut_. Assuming that the probability that *n_j_* > *n*_cut_ is small enough to be neglected, normalizing the joint probabilities to 1 provides a good approximation to the conditional distribution. Calculating these joint probabilities dominates the computational effort for sampling each mode and grows with the number of detected photons.

We find that the joint probabilities are given by

$$P(n_{1},\ldots ,n_{m},\beta_{m+1},\ldots ,\beta_{M})\propto \frac{{|\text{lhaf}\;(B_{\vec{n},\vec{\beta }})|}^{2}}{n_{m}!}$$ (11)

where $B_{\vec{n},\vec{\beta }}$ is formed from ***B*** by repeating the *i*th row and column *n_i_* times and then, in the same manner, repeating the entries of γ′ along the diagonal of $B_{\vec{n},\vec{\beta }}$, where γ′ is given by

$$\gamma \prime ={\left(\vec{\alpha }-\vec{\beta }\right)}^{†}\sigma_{Q}^{-1}$$ (12)

Here, $\vec{n}$ is nonzero only for the modes that have already been sampled in photon number, and similarly, the values of $\vec{\beta }$ are set to zero as the corresponding mode is sampled in photon number. We note that because *n*_cut_ should usually be several times greater than the expected number of photons, these calculations will often contain photon collisions.

To arrive at Eqs. 11 and 12, we note that the projection of the output state ∣ψ⟩ onto the state ∣*n*_1_, …, *n_m_*, β_*m*+1_, …, β*_M_*⟩ can be expressed as

$$\begin{array}{c} \langle n_{1},\ldots ,n_{m},\beta_{m+1},\ldots ,\beta_{M}|\psi \rangle \\ =\langle n_{1},\ldots ,n_{m},0,\ldots ,0|{\hat{D}}^{†}(\vec{\beta })|\psi \rangle \\ =\langle n_{1},\ldots ,n_{m},0,\ldots ,0|\hat{D}(-\vec{\beta })|\psi \rangle \end{array}$$ (13)

where $\hat{D}(\vec{\beta })$ is the displacement operator. Hence, we can apply a displacement of $-\vec{\beta }$ to the state and calculate the probability from a projection onto a photon number state, with vacuum in the modes that are yet to be sampled.

In the Supplementary Materials, we describe algorithms to speed up loop hafnian calculations in the presence of detecting photon collision events and a method of batching together the calculations for different *n_j_* such that the total run time is approximately equal to that of calculating the largest *n_j_*. When simulating threshold detectors, we expand each mode to several subdetectors, as shown in Fig. 1E, and treat them as separate modes in the chain rule sampling algorithm, with the only difference being that once a photon is detected, no further information is required from the remaining subdetectors within that mode. Hence, they can continue to be projected onto the coherent state basis, where they do not contribute to the complexity of calculating the probabilities. In the Supplementary Materials, we discuss a batched method of calculating the loop hafnians required for different subdetectors within the same mode, achieving a speedup by noting that only the diagonal entries of $B_{\vec{n},\vec{\beta }}$ change between subdetectors.

Because the order with which this algorithm progresses through the modes is arbitrary, we choose to go in order of increasing mean photon/click number. This slightly reduces the run time because photons are less likely to be detected in the earlier modes, so the size of the loop hafnians required in these stages is generally reduced. An implementation of the chain rule algorithm and all other algorithms presented in this work can be found in our online code repository (*42*).

## Supplementary Materials

### This PDF file includes:

Supplementary Text Figs. S1 to S13 Table S1 References

## References

[1] S. Aaronson, A. Arkhipov, The computational complexity of linear optics, in *Proceedings of the Forty-Third Annual ACM Symposium on Theory of Computing* (ACM, 2011), pp. 333–342.

[2] A. P. Lund, A. Laing, S. Rahimi-Keshari, T. Rudolph, J. L. O’Brien, T. C. Ralph, Boson sampling from a Gaussian state. Phys. Rev. Lett. 113, 100502 (2014). 25238340 10.1103/PhysRevLett.113.100502

[3] C. S. Hamilton, R. Kruse, L. Sansoni, S. Barkhofen, C. Silberhorn, I. Jex, Gaussian boson sampling. Phys. Rev. Lett. 119, 170501 (2017). 29219463 10.1103/PhysRevLett.119.170501

[4] R. Kruse, C. S. Hamilton, L. Sansoni, S. Barkhofen, C. Silberhorn, I. Jex, Detailed study of Gaussian boson sampling. Phys. Rev. A 100, 032326 (2019). 10.1103/PhysRevLett.119.170501 29219463

[5] N. Quesada, Franck-Condon factors by counting perfect matchings of graphs with loops. J. Chem. Phys. 150, 164113 (2019). 31042916 10.1063/1.5086387

[6] H.-S. Zhong, H. Wang, Y.-H. Deng, M.-C. Chen, L.-C. Peng, Y.-H. Luo, J. Qin, D. Wu, X. Ding, Y. Hu, P. Hu, X.-Y. Yang, W.-J. Zhang, H. Li, Y. Li, X. Jiang, L. Gan, G. Yang, L. You, Z. Wang, L. Li, N.-L. Liu, C.-Y. Lu, J.-W. Pan, Quantum computational advantage using photons. Science 370, 1460–1463 (2020). 33273064 10.1126/science.abe8770

[7] N. Quesada, J. M. Arrazola, N. Killoran, Gaussian boson sampling using threshold detectors. Phys. Rev. A 98, 062322 (2018).

[8] D. Grier, D. J. Brod, J. M. Arrazola, M. B. de Andre Alonso, N. Quesada, The complexity of bipartite Gaussian boson sampling. arXiv:2110.06964 [quant-ph] (13 October 2021).

[9] N. Quesada, R. S. Chadwick, B. A. Bell, J. M. Arrazola, T. Vincent, H. Qi, R. García-Patrón, Quadratic speedup for simulating Gaussian boson sampling. arXiv:2010.15595 [quant-ph] (29 October 2020).

[10] A. Neville, C. Sparrow, R. Clifford, E. Johnston, P. M. Birchall, A. Montanaro, A. Laing, Classical boson sampling algorithms with superior performance to nearterm experiments. Nat. Phys. 13, 1153–1157 (2017).

[11] N. Quesada, J. M. Arrazola, Exact simulation of Gaussian boson sampling in polynomial space and exponential time. Phys. Rev. Res. 2, 023005 (2020).

[12] B. Wu, B. Cheng, F. Jia, J. Zhang, M.-H. Yung, X. Sun, Speedup in classical simulation of Gaussian boson sampling. Sci. Bull. 65, 832–841 (2020). 10.1016/j.scib.2020.02.012 36659202

[13] B. Gupt, J. M. Arrazola, N. Quesada, T. R. Bromley, Classical benchmarking of Gaussian Boson Sampling on the Titan supercomputer. Quantum Inf. Process 19, 249 (2020).

[14] A. Serafini, *Quantum Continuous Variables* (CRC Press, 2017).

[15] H. J. Ryser, *Combinatorial Mathematics* (Mathematical Association of America, 1963), vol. 14.

[16] V. S. Shchesnovich, Asymptotic evaluation of bosonic probability amplitudes in linear unitary networks in the case of large number of bosons. Int. J. Quantum Inf. 11, 1350045 (2013).

[17] M. Tichy, “Entanglement and interference of identical particles,” thesis, Albert-Ludwigs-Universität Freiburg, Freiburg (2011).

[18] S. Chin, J. Huh, Generalized concurrence in boson sampling. Sci. Rep. 8, 6101 (2018). 29666423 10.1038/s41598-018-24302-5 PMC5904218

[19] R. Kan, From moments of sum to moments of product. J. Multivar. Anal. 99, 542–554 (2008).

[20] A. Björklund, B. Gupt, N. Quesada, A faster hafnian formula for complex matrices and its benchmarking on a supercomputer. J. Exp. Algorithmics 24, 1–17 (2019).

[21] M. Cygan, M. Pilipczuk, Faster exponential-time algorithms in graphs of bounded average degree. Inf. Comput. 243, 75–85 (2015).

[22] K. Balasubramanian, “Combinatorics and diagonals of matrices,” thesis, Indian Statistical Institute, Calcutta (1980).

[23] E. Bax, J. Franklin, A finite-difference sieve to count paths and cycles by length. Inf. Process. Lett. 60, 171–176 (1996).

[24] D. G. Glynn, The permanent of a square matrix. Eur. J. Comb. 31, 1887–1891 (2010).

[25] O. F. Thomas, W. McCutcheon, D. P. S. McCutcheon, A general framework for multimode Gaussian quantum optics and photo-detection: Application to Hong–Ou–Mandel interference with filtered heralded single photon sources. APL Photonics 6, 040801 (2021).

[26] J. S. Liu, Metropolized independent sampling with comparisons to rejection sampling and importance sampling. Stat. Comput. 6, 113–119 (1996).

[27] J. S. Liu, *Monte Carlo Strategies in Scientific Computing* (Springer Publishing Company Incorporated, 2008).

[28] L. Devroye, Nonuniform random variate generation, in *Handbooks in Operations Research and Management Science*, S. G. Henderson, B. L. Nelson, Eds. (Elsevier, 2006), vol. 13, pp. 83–122.

[29] F. Mezzadri, How to generate random matrices from the classical compact groups. Not. Am. Math. Soc 54, 592–604 (2006).

[30] H.-S. Zhong, Y.-H. Deng, J. Qin, H. Wang, M.-C. Chen, L.-C. Peng, Y.-H. Luo, D. Wu, S.-Q. Gong, H. Su, Y. Hu, P. Hu, X.-Y. Yang, W.-J. Zhang, H. Li, Y. Li, X. Jiang, L. Gan, G. Yang, L. You, Z. Wang, L. Li, N.-L. Liu, J.-J. Renema, C.-Y. Lu, J.-W. Pan, Phase-programmable Gaussian boson sampling using stimulated squeezed light. Phys. Rev. Lett. 127, 180502 (2021). 34767431 10.1103/PhysRevLett.127.180502

[31] B. Villalonga, M. Y. Niu, L. Li, H. Neven, J. C. Platt, V. N. Smelyanskiy, S. Boixo, Efficient approximation of experimental Gaussian boson sampling. arXiv:2109.11525 [quant-ph] (23 September 2021).

[32] J. J. Renema, A. Menssen, W. R. Clements, G. Triginer, W. S. Kolthammer, I. A. Walmsley, Efficient classical algorithm for boson sampling with partially distinguishable photons. Phys. Rev. Lett. 120, 220502 (2018). 29906153 10.1103/PhysRevLett.120.220502

[33] A. E. Moylett, R. García-Patrón, J. J. Renema, P. S. Turner, Classically simulating near-term partially-distinguishable and lossy boson sampling. Quantum Sci. Technol. 5, 015001 (2020).

[34] R. García-Patrón, J. J. Renema, V. Shchesnovich, Simulating boson sampling in lossy architectures. Quantum 3, 169 (2019).

[35] D. J. Brod, M. Oszmaniec, Classical simulation of linear optics subject to nonuniform losses. Quantum 4, 267 (2020).

[36] J. Shi, T. Byrnes, Gaussian boson sampling with partial distinguishability. arXiv:2105.09583 [quant-ph] (20 May 2021).

[37] N. Quesada, L. G. Helt, J. Izaac, J. M. Arrazola, R. Shahrokhshahi, C. R. Myers, K. K. Sabapathy, Simulating realistic non-Gaussian state preparation. Phys. Rev. A 100, 022341 (2019).

[38] C. Weedbrook, S. Pirandola, R. García-Patrón, N. J. Cerf, T. C. Ralph, J. H. Shapiro, S. Lloyd, Gaussian quantum information. Rev. Mod. Phys. 84, 621–669 (2012).

[39] S. Olivares, Quantum optics in the phase space. Eur. Phys. J. Spec. Top. 203, 3–24 (2012).

[40] J. E. Gentle, *Computational Statistics* (Springer, 2009), vol. 308.

[41] P. Clifford, R. Clifford, The classical complexity of boson sampling, in *Proceedings of the Twenty-Ninth Annual ACM-SIAM Symposium on Discrete Algorithms* (SIAM, 2018), pp. 146–155.

[42] https://github.com/jakeffbulmer/gbs.

[43] B. Gupt, J. Izaac, N. Quesada, The Walrus: A library for the calculation of hafnians, Hermite polynomials and Gaussian boson sampling. J. Open Source Softw. 4, 1705 (2019).

[44] S. K. Lam, A. Pitrou, S. Seibert, Numba: A llvmbased python jit compiler, in *Proceedings of the Second Workshop on the LLVM Compiler Infrastructure in HPC* (Association for Computing Machinery, 2015), pp. 1–6.

[45] L. Dalcín, R. Paz, M. Storti, J. D’Elía, MPI for Python: Performance improvements and MPI-2 extensions. J. Parallel Distrib. Comput. 68, 655–662 (2008).

[46] L. Latmiral, N. Spagnolo, F. Sciarrino, Towards quantum supremacy with lossy scattershot boson sampling. New J. Phys. 18, 113008 (2016).

[47] P. Clifford, R. Clifford, Faster classical Boson Sampling. arXiv:2005.04214 [quant-ph] (7 May 2020).

[48] M. Rudelson, A. Samorodnitsky, O. Zeitouni, Hafnians, perfect matchings and Gaussian matrices. Ann. Probab. 44, 2858–2888 (2016).

[49] D. S. Phillips, M. Walschaers, J. J. Renema, I. A. Walmsley, N. Treps, J. Sperling, Benchmarking of Gaussian boson sampling using two-point correlators. Phys. Rev. A 99, 023836 (2019).

