# Supplementary material for: The boundary for quantum advantage in Gaussian boson sampling
Source: Sci Adv. 2022 Jan 26;8(4):eabl9236. doi: 10.1126/sciadv.abl9236 (PMC8791606; doi:10.1126/sciadv.abl9236)
Supplement: Supplementary file 1 — Supplementary Text Figs. S1 to S13 Table S1 References [file sciadv.abl9236_sm.pdf]

Science Advances
AAAS

Supplementary Materials for

The boundary for quantum advantage in Gaussian boson sampling

Jacob F. F. Bulmer*, Bryn A. Bell*, Rachel S. Chadwick, Alex E. Jones, Diana Moise, Alessandro Rigazzi, Jan Thorbecke, Utz-Uwe Haus, Thomas Van Vaerenbergh, Raj B. Patel, Ian A. Walmsley, Anthony Laing*

*Corresponding author. Email: jacob.bulmer@bristol.ac.uk (J.F.F.B.); b.bell@imperial.ac.uk (B.A.B.); anthony.laing@bristol.ac.uk (A.L.)

Published 26 January 2022, *Sci. Adv.* **8**, eabl9236 (2022)
DOI: 10.1126/sciadv.abl9236

This PDF file includes:

Supplementary Text
Figs. S1 to S13
Table S1
References

CONTENTS

1. Loop hafnian algorithms 2
   A. Eigenvalue-trace 2
   B. Repeated pairs 3
   C. Matching algorithm 4
   D. Finite difference sieve 4
   E. Batching probability calculations 6
   F. Implementation details 6

2. Exact simulation algorithms 6
   A. Boson Sampling 6
   B. Gaussian Boson Sampling 6

3. MIS GBS algorithms 8
   A. Independent Pairs and Singles GBS distribution 8
   B. PNRD GBS 8
   C. Threshold detector GBS 9
   D. Thinning interval and burn-in time scaling 11

4. Validation tests 15
   A. CHOG ratio 15
      1. IPS vs thermal 16
      2. MIS vs IPS 16
   B. Output correlations 16

1. LOOP HAFNIAN ALGORITHMS

The loop hafnian function of an $N \times N$ symmetric matrix $\boldsymbol{A}$ is defined as

$$\mathrm{lhaf}(\boldsymbol{A}) = \sum_{M \in \mathrm{SPM}} \prod_{(i,j) \in M} A_{ij}, \tag{S1}$$

where SPM is the set of single-pair matchings, the ways in which the indices $[N]$ can be grouped into sets of sizes 1 and 2, and A_{ij} are the elements of $\boldsymbol{A}$. This is a generalisation of the set of perfect matchings (all of the groupings into pairs) which defines the a hafnian, but with additional 'loops' referring to sets of size 1. These loops have weightings given by the diagonal elements of the matrix.

A. Eigenvalue-trace

The eigenvalue-trace algorithm for the loop hafnian (with N even[1]) can be written as [20]:

$$\mathrm{lhaf}(\boldsymbol{A}) = \sum_{\vec{z}} \left(\prod_{k=1}^{N/2} (-1)^{z_k} \right) f\left(\boldsymbol{A}, \vec{z}\right). \tag{S2}$$

where $z_i \in \{0,1\}$ and $\vec{z}$ describes all possible $N/2$ length binary strings.

The function $f\left(\boldsymbol{A}, \vec{z}\right)$ is defined as the $\lambda^{N/2}$ coefficient of the polynomial:

$$p_{N/2}(\lambda, \boldsymbol{A}, \vec{z}) = \sum_{j=1}^{N/2} \frac{1}{j!} \left(\sum_{k=1}^{N/2} \left(\frac{\mathrm{Tr}((\boldsymbol{A}\boldsymbol{X}_{\vec{z}})^k)}{2k} + \frac{\vec{v}\boldsymbol{X}_{\vec{z}}(\boldsymbol{A}\boldsymbol{X}_{\vec{z}})^{k-1}\vec{v}^T}{2} \right) \lambda^k \right)^j \tag{S3}$$

---

[1] For odd N, one can either pad $\boldsymbol{A}$ with an additional row/column [43], or modify the function f to account for a single unpaired edge. In our code [42], we opt to modify f.

where $\vec{v}$ is a vector given by the diagonal elements of $\boldsymbol{A}$, and $\boldsymbol{X}_{\vec{z}}$ is defined like $\boldsymbol{X}$, introduced in the main text, but with the 1s on the off-diagonal blocks replaced by $\vec{z}$:

$$\boldsymbol{X}_{\vec{z}} = \begin{pmatrix} \mathbf{0} & \mathrm{diag}(\vec{z}) \\ \mathrm{diag}(\vec{z}) & \mathbf{0} \end{pmatrix}. \tag{S4}$$

There are rows and columns of $\boldsymbol{A}\boldsymbol{X}_{\vec{z}}$ which are all 0s. These rows can be removed to speed up the evaluation of f.

The eigenvalue-trace algorithm can be thought of as performing inclusion/exclusion over the set of pairs in one fixed perfect matching, defined by $\boldsymbol{X}$. A derivation of this formula can be found in ref. [20], however note that equation 25 and 26 of ref. [20] differs as we apply the matrix multiplication by $\boldsymbol{X}_{\vec{z}}$ inside the function f, whereas ref. [20] do this before passing the matrix to this function. This change in notation allows us to generalise this function in the next section.

The complexity of evaluating f is dominated by finding the traces of matrix powers, $\mathrm{Tr}(\boldsymbol{A}\boldsymbol{X}_{\vec{z}})^k$, which can be reduced to finding the eigenvalues of $(\boldsymbol{A}\boldsymbol{X}_{\vec{z}})$ in $\mathcal{O}(N^3)$ time. Once we find the eigenvalues of $(\boldsymbol{A}\boldsymbol{X}_{\vec{z}})$, given by $\{e_i\}$, we can compute trace powers as: $\mathrm{Tr}((\boldsymbol{A}\boldsymbol{X}_{\vec{z}})^k) = \sum_i e_i^k$. There are $2^{N/2}$ terms in the summation in equation S2, so this results in $\mathcal{O}(N^3 2^{N/2})$ complexity.

B. Repeated pairs

This algorithm makes use of a fixed perfect matching given by the adjacency matrix $\boldsymbol{X}$, defining pairs $(i, N/2+i)$ for $i \in [1, N/2)$. The summation in equation S2 corresponds to inclusion/exclusion of these pairs. If we consider the way that the $\boldsymbol{A}_{\vec{n}}$ matrix is formed when evaluating the $\vec{n}$ probability from a mixed state (described in the "multiple photons in the same mode" section of ref. [4]), $\boldsymbol{X}$ will pair the ith index in $\boldsymbol{A}$ with the $(i+M)$th index, and this pairing will be repeated n_i times. Instead of summing over all $z_i \in \{0,1\}$, where each z_i represents a different photon, we can instead let $z_i \in \{0,1,\ldots,n_i\}$, corresponding to including z_i copies of the ith pair:

$$\mathrm{lhafmix}(\boldsymbol{A}, \boldsymbol{\gamma}, \vec{n}) = \sum_{\vec{z}} (-1)^{|\vec{z}|} \prod_i \binom{n_i}{z_i} f'(\boldsymbol{A}, \boldsymbol{\gamma}, \vec{z}). \tag{S5}$$

We label this function lhafmix because it does not apply to general matrices, only those with the particular form of $\boldsymbol{A}_{\vec{n}}$. $f'(\boldsymbol{A}, \boldsymbol{\gamma}, \vec{z})$ is defined as the $\lambda^{N/2}$ coefficient in the polynomial:

$$p'_{N/2}(\lambda, \boldsymbol{A}, \boldsymbol{\gamma}, \vec{z}) = \sum_{j=1}^{N/2} \frac{1}{j!} \left(\sum_{k=1}^{N/2} \left(\frac{\mathrm{Tr}((\boldsymbol{A}\boldsymbol{X}_{\vec{z}})^k)}{2k} + \frac{\boldsymbol{\gamma}\boldsymbol{X}_{\vec{z}}(\boldsymbol{A}\boldsymbol{X}_{\vec{z}})^{k-1}\boldsymbol{\gamma}^T}{2} \right) \lambda^k \right)^j. \tag{S6}$$

The polynomials p' and p are almost identical, but in order to correctly construct the matrix associated with multiple photons arriving in the same mode, we must pass through $\boldsymbol{\gamma}$ explicitly, instead of inferring it from the diagonal elements of $\boldsymbol{A}$. Details can be found in the "multiple bosons in the same mode" section of ref. [5].

Equation S5 makes use of the fact that increasing the weight of a pairing in $\boldsymbol{X}$ has the same effect as including a pair multiple times. This algorithm calculates mixed state probabilities in time $\mathcal{O}\left(N^3 \prod_i (n_i+1)\right)$. Noting that this corresponds to a $2N \times 2N$ loop hafnian, this compares well to using the repeated moment algorithm [19], which would take $\mathcal{O}\left(N \prod_i (n_i+1)^2\right)$, and improves on eigenvalue-trace whenever there are elements of $\vec{n}$ greater than 1.

For general matrices such as those in pure state calculations, even if there are photon collisions which lead to repeated rows/columns in the B matrix, these do not necessarily lead to repeated pairings. However if identical pairs do occur, with the ith pair occurring η_i times, we can make use of the above formula to obtain some speed-up, reducing the number of inclusion/exclusion terms to $\prod_i (\eta_i + 1)$. This quantity is upper-bounded by $2^{N/2}$, which occurs if no pairs are repeated. For even total photon number, it is lower-bounded by $\prod_j \sqrt{n_j+1}$. To see this, consider that for a total of H unique pairings we can write:

$$\prod_{i=1}^{H} (\eta_i + 1) = \prod_{i=1}^{H} \prod_{j=1}^{M} \prod_{k=L,R} \sqrt{n_j^{(i,k)} + 1}, \tag{S7}$$

with $n_j^{(i,k)}$ the number of photons from mode j which are associated with the ith pair and position $k = L, R$ within that pair. The equality follows from the fact that only one mode will be associated with a particular (i,k), i.e. for a

given (i,k) there is only one j for which $n_j^{(i,k)}$ is non-zero. The factor associated with a given mode j is lower-bounded:

$$\prod_{i=1}^{H}\prod_{k=L,R}\sqrt{n_j^{(i,k)}+1}\geq\sqrt{n_j+1}, \tag{S8}$$

which occurs when $n_j^{(i,k)}$ is non-zero for only one choice of (i,k). Hence the overall number of inclusion/exclusion terms is lower-bounded by

$$\prod_{i=1}^{H}(\eta_i+1)\geq\prod_{j=1}^{M}\sqrt{n_j+1}. \tag{S9}$$

C. Matching algorithm

Since the fixed perfect matching in the eigenvalue-trace algorithm is arbitrary, we can choose it so as to create identical pairs and reduce the number of steps. Equivalently, we can permute rows/columns in the input matrix so as to change the pairings created using the X matrix. Here, we give a greedy algorithm which chooses the pairings in the fixed perfect matching so as to minimise the number of inclusion/exclusion steps.

The algorithm takes as input the vector of photon numbers $\vec{n}$. We start by creating a list of mode labels $\vec{m}=(0,1,\ldots,M-1)$. This algorithm returns a list of mode pairings, $\vec{P}$, and how many times each pairing is repeated, $\vec{\eta}$. The algorithm then proceeds as follows:

1. Sort the modes in decreasing order of photon number, i.e. sort $\vec{n}$ and $\vec{m}$ according to $\vec{n}$
2. If $n_1\geq 2n_2$: append $\lfloor n_1/2\rfloor$ to $\vec{\eta}$ and (m_1,m_1) to P. Subtract $2\lfloor n_1/2\rfloor$ from n_1
   Else: append n_2 to $\vec{\eta}$ and (m_1,m_2) to $\vec{P}$. Subtract n_2 from both n_1 and n_2.
3. Remove elements n_i and m_i from $\vec{n},\vec{m}$ for all $n_i=0$
4. If $\sum\vec{n}>1$: return to step 1
   Else: return $\vec{P}$, $\vec{\eta}$ and, for odd total photon number, the remaining element of $\vec{m}$

Here we use $\lfloor\cdot\rfloor$ to notate rounding down to the nearest integer.

An implementation of this algorithm can be found in the function `matched_reps` of our repository [42]. In Fig. **S1**, we show that this algorithm manages to achieve comparable scaling to the lower bound in equation S9, and all examples investigated are within a factor of 3 of this lower bound. Following the repeated pairs algorithm above, the loop hafnian can be calculated in time $\mathcal{O}\left(N^3\prod(\eta_i+1)\right)$. This improves on eigenvalue-trace for a general matrix whenever $\vec{\eta}$ contains an entry >1, and this is true whenever there are at least two elements >1 in $\vec{n}$, or if there is at least one element ≥ 4.

D. Finite difference sieve

By analogy to the Glynn formula for the permanent [22–24], we can find an alternative expression for the loop hafnian which uses a finite difference sieve instead of an inclusion/exclusion formula:

$$\text{lhaf}(A)=\frac{1}{2^{N/2}}\sum_{\vec{\delta}}\left(\prod_{k=1}^{N/2}\delta_k\right)f(AX_{\vec{\delta}}) \tag{S10}$$

where $\vec{\delta}$ describes all possible $N/2$ length vectors with $\delta_i\in\{-1,1\}$. Here $X_{\vec{\delta}}$ is defined as:

$$X_{\vec{\delta}}=\begin{pmatrix}\mathbf{0} & \text{diag}(\vec{\delta})\\ \text{diag}(\vec{\delta}) & \mathbf{0}\end{pmatrix}. \tag{S11}$$

Since an overall sign change to $\vec{\delta}$ leaves the terms inside the summation unchanged, one element of $\vec{\delta}$ can be fixed, e.g. $\delta_1=1$, and the result multiplied by 2, halving the run-time. One way to think of the permanent or the loop

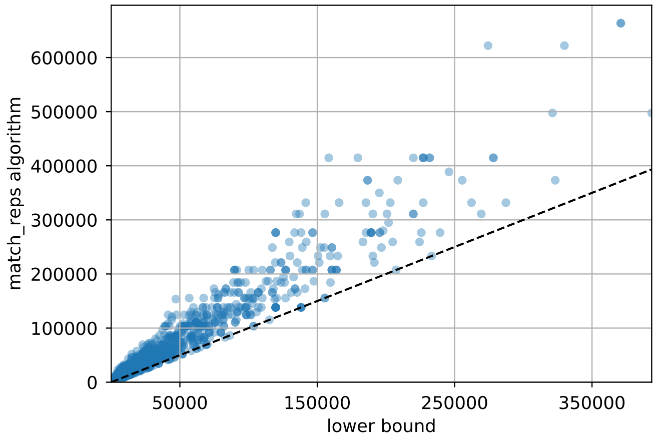

Supplementary Figure S1. **Numerical benchmarking of edge matching algorithm.** Number of terms in loop hafnian sum, estimated using lower bound and computed using the `matched_reps` algorithm reported in section 1 C. This is carried out for a range of even total photon numbers up to $N = 30$ and a range of number of modes up to $M = 50$, with measurement outcome sampled from a uniform distribution. Black dashed line shows this bound, and we note that all samples fall above this line.

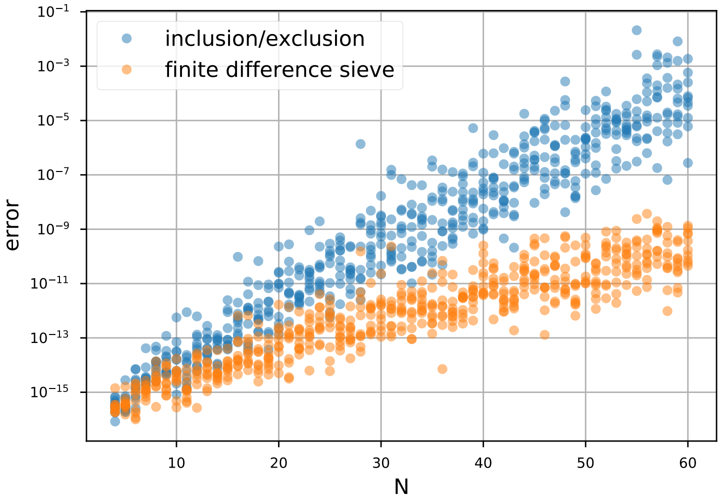

Supplementary Figure S2. **Numerical precision of finite difference sieve and inclusion/exclusion loop hafnian algorithms.** We construct an $N \times N$ matrix, C, using 2 random $N/2 \times N/2$ matrices, A and B, on the diagonal quadrants. We define: error $= |\frac{\mathrm{lhaf}(C) - \mathrm{lhaf}(A)\,\mathrm{lhaf}(B)}{\mathrm{lhaf}(A)\,\mathrm{lhaf}(B)}|$. We plot the error for 10 random instances for each N using 64 nodes of the Isambard HPC system.

hafnian is that it represents the coefficient of some polynomial. Calculating this coefficient can be done by evaluating derivatives of this polynomial at the origin. In this picture, equation S2 corresponds to a forward difference derivative, and equation S10 corresponds to a central difference derivative [23]

We can make use of repeated pairings in the finite difference sieve algorithm in the same way as above. Then, for each pairing, δ runs from $-n_{\mathrm{pair}}$ to $+n_{\mathrm{pair}}$ in steps of 2, with the different terms corresponding to how many copies of the pair are associated with a -1 or $+1$.

We find that this method offers significant accuracy improvements over inclusion/exclusion, as shown in Fig. **S2**. In both algorithms, the absolute value of the machine precision errors accumulate at a fairly similar rate inside the sum, however in the finite difference sieve, the prefactor in equation S10 divides the value of the error by the number of terms in the sum.

E. Batching probability calculations

For each step of the chain-rule algorithm, we require probabilities where all but one mode has a fixed outcome, $\vec{n}_{\text{fixed}}$, while one 'batched' mode takes all values from 0 to the cutoff, n_{cut}.

In the pair matching algorithm, we only input $\vec{n}_{\text{fixed}}$. If we consider the calculation when the batched mode is equal to n_{cut}, this leaves $\lfloor n_{n_{\text{cut}}}/2 \rfloor$ copies of the batched mode paired to itself. Whilst computing the probability for the n_{cut} outcome, we will also compute all the necessary eigenvalues required for calculating any outcome $\leq n_{n_{\text{cut}}}$. Since finding these eigenvalues is the only cubic time step within each term in the sum, we can compute all probabilities for $n \leq n_{n_{\text{cut}}}$ in the same time complexity as calculating $P(\vec{n}_{\text{fixed}}, n_{n_{\text{cut}}})$. For batching across sub-detectors within the same mode in threshold detector sampling, each detector is treated independently and so has a different β_i, but this does not change the eigenvalue calculation, so these calculations can be batched in a similar way. We have implemented these methods in [42].

These methods could also be applied to speed up calculations of heralded non-Gaussian states in the Fock basis, as is described in ref. [37].

F. Implementation details

Our loop hafnian code is written in Python and uses Numba, a just-in-time compiler which automatically generates highly efficient code [44]. To run efficiently on distributed systems, we use MPI for Python [45]. The eigenvalue-trace algorithm is readily parallelisable as each term in the sum can be computed independently of all other terms.

Whilst testing and benchmarking our code, we ran on all major operating systems, and on x86-64 and arm64 architectures. Both Fugaku and the Isambard system (used for data in Fig. **S2**) use arm64 chip architectures, giving us further confidence in our run-time predictions.

2. EXACT SIMULATION ALGORITHMS

A. Boson Sampling

During the early development of boson sampling, it was sometimes assumed that to simulate Boson sampling experiments, the full probability distribution must be computed [46]. It was later shown that approximate sampling methods, such as Metropolis Independence Sampling (MIS) and rejection sampling could be used to simulate boson sampling, without a 'brute-force' computation of the probability distribution [10]. Soon after, a probability chain-rule method was discovered which allowed for exact sampling [41]. This algorithm was subsequently updated to include speedups due to collisions [47] by using known results on computing permanents with repeated rows [16–18].

B. Gaussian Boson Sampling

The first reported simulation algorithm for GBS with number resolving detectors which improved upon the brute-force approach was a chain-rule based algorithm which calculates mixed-state measurement probabilities by Quesada et al. [11]. Two alternate chain-rule approaches were reported shortly after by Wu et al. [12]. A quadratic speedup to ref. [11] was recently reported [9] which proposes a method where only pure-state probabilities are required.

Although not reported in the literature, an implementation of ref. [11] in The Walrus libary [43] uses the repeated moment hafnian algorithm [19], when collisions sufficiently dominate for this approach to be faster than eigenvalue-trace (without exploiting collisions).

For Gaussian Boson Sampling with threshold detectors, one can simulate number resolving detectors then record any outcomes with $n \geq 1$ as a click. However, algorithms tailored to threshold detector GBS have also been reported. An exponential time, exponential space algorithm was used to simulate up to 20 click experiments [13]. However, a chain-rule method using the Torontonian has been proposed [11]. The time complexity of these algorithms is shown in table **S1**.

To visualise how these different algorithms establish a boundary for quantum advantage, we approximately estimate the required size of experiment for the runtime on Fugaku, the world's fastest supercomputer, to reach 1 day. This is shown in Fig. 1 F. However, we wish to emphasise that the choice of 1 day per sample is arbitrary and also that an experiment placing within the region of quantum advantage in this figure would not make for sufficient evidence to claim quantum advantage alone. It is also required to show evidence that the samples could not have been generated

by faster, approximate algorithms. Without such evidence, errors from an experiment could lead to errors in the probability distribution which remove the computational complexity.

**Algorithm**	**Time complexity**
Collision-free PNRD	
Chain-rule mixed [11]	$MN^3 2^N$
Wu et al. (1) [12]	$\text{poly}(N) 2^{8N/2}$
Wu et al. (2) [12]	$\text{poly}(N) 2^{5N/2} \dagger$
Chain-rule pure [9]	$MN^3 2^{N/2}$
Collisions PNRD	
Chain-rule mixed, rep. mom. [11, 19, 43]	MNG^N
This work	$MN^3 G^{N/2}$
Threshold detector	
Threshold [7]	$M 2^{N_c} \dagger$
Chain-rule mixed [11]	$MN^3 2^{N_c}$
This work	$MN^3 2^{N_c/2}$

Supplementary Table S1. **Exact GBS simulation methods $\mathcal{O}$ time complexity comparison.** Collision-free PNRD algorithms are given for the complexity to sample an event where N photons are detected over M modes. PNRD with collisions are given for M mode, N photon events, where detector outcomes have a parameter, $G = \left(\prod_i^M (n_i + 1)\right)^{1/N}$. To allow for comparison, we report our work using the lower bound reported in the main text. Threshold detector algorithms are given for M mode experiments detecting N_c clicks. Algorithms with exponential space complexity are indicated with (†) symbol.

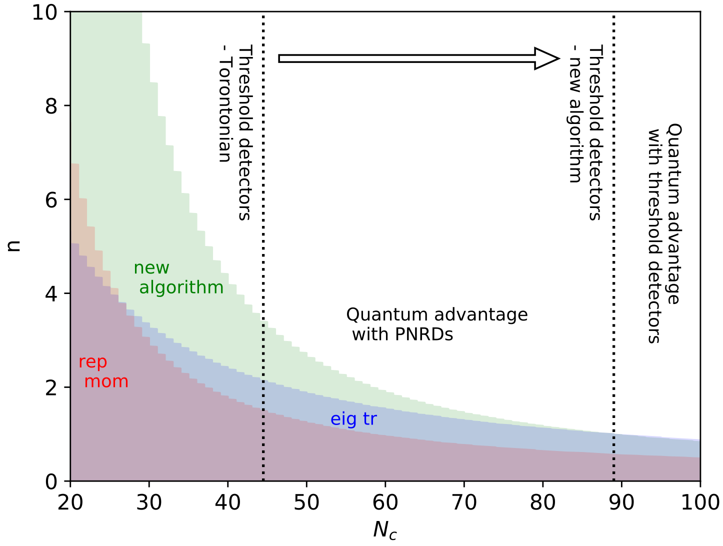

Supplementary Figure S3. **Boundary for quantum advantage using different simulation algorithms.** The boundary is chosen as the estimated experimental scale for the simulation time on Fugaku, the world's fastest supercomputer, to calculate a single probability. To allow comparison between different algorithms, we consider the sample to calculate the probability of an event where N_c detectors each measure n photons. Black dashed lines show this boundary when using threshold detectors, using the Torontonian function [7] or the algorithm presented in this work. The repeated moment (red) [19, 43], eigenvalue-trace (blue) [20] and our results (green) for loop hafnians are compared to show the boundary for PNRDs.

3. MIS GBS ALGORITHMS

MIS is a Markov Chain Monte Carlo method of sampling which works by suggesting a state from a proposal distribution and accepting it according to the acceptance probability, equation 3. Otherwise, the previous state is added to the Markov chain.

A. Independent Pairs and Singles GBS distribution

Choosing a suitable proposal distribution is an extremely important factor for MIS to be useful. If the proposal distribution does not match closely to the target distribution, this will result in low acceptance probabilities, and hence a very long thinning interval. Here, we introduce an Independent Pairs and Singles (IPS) distribution, where as the name suggests we generate multi-photon samples from many independent single-photon and pair-photon generation processes, without quantum interference between separately generated singles/pairs. We find this is a better approximation to GBS than other efficiently simulable alternatives such as thermal states or distinguishable squeezed states.

Beginning from a pure Gaussian state which we wish to approximate, we first sample the number of individual photons created in each mode by the displacement, using Poisson distributions with the mean of the jth mode given by $|\alpha_j|^2$. We then sample the number of photon pairs created by squeezing between all mode pairs (j, k) (with $j \leq k$) from a Poisson distribution with mean given by $|\boldsymbol{B}|^2_{j,k}$. Combining all outcomes results in a photon number pattern, $\vec{n}$.

For MIS, we must calculate the probability of our generated proposal sample, $\vec{n}$. There are many possible ways to create the same sample, corresponding to different groupings of the photons into pairs and singles. The total probability is related to a loop hafnian, which contains a corresponding sum over all single-pair matchings. We can write this probability as:

$$Q(\vec{n}|\boldsymbol{B}, \alpha) = \frac{e^{-\sum_j |\alpha_j|^2} e^{-\sum_{j,k} \frac{1}{2}|B_{j,k}|^2}}{\prod_i n_i!} \operatorname{lhaf}(\boldsymbol{C}_{\vec{n}}), \tag{S12}$$

where $\boldsymbol{C}_{\vec{n}}$ is the matrix formed by taking $|\boldsymbol{B}_{\vec{n}}|^2$ and replacing the diagonal elements with $|\vec{\alpha}_{\vec{n}}|^2$.

The loop hafnian of a positive matrix is likely to be efficient to compute approximately [43, 48]. However, in MIS we must also compute a loop hafnian of a complex matrix to evaluate the target probability of the sample. Hence for convenience and simplicity we make use of the same optimised and parallelised code to compute both loop hafnians, without losing any accuracy. This increases the run-time by at most a factor of 2.

B. PNRD GBS

We first consider the case of sampling in the photon number basis, $\vec{n}$. We expand the sample space to include a displacement variable, $\vec{\alpha}$, so that only pure-state probabilities need to be evaluated. The target distribution $P(\vec{n}, \vec{\alpha})$ can be written as $P(\vec{\alpha})P(\vec{n}|\vec{\alpha})$ where $P(\vec{\alpha})$ is a multivariate normal distribution and hence efficient to sample from, while $P(\vec{n}|\vec{\alpha})$ is given by equation 7 and depends on an $N \times N$ loop hafnian. We choose $Q(\vec{\alpha}) = P(\vec{\alpha})$, which results in the acceptance probability:

$$p_{\text{accept}} = \min\left(1, \frac{P(\vec{n}_i|\vec{\alpha}_i)Q(\vec{n}_{i-1}|\vec{\alpha}_{i-1})}{P(\vec{n}_{i-1}|\vec{\alpha}_{i-1})Q(\vec{n}_i|\vec{\alpha}_i)}\right), \tag{S13}$$

so the acceptance probability does not depend on the probability density of $\vec{\alpha}$.

In some cases it may be useful to fix the total photon number N when sampling; for example verification methods often focus on samples of a particular N. In MIS it is possible to fix N by post-selecting our proposed states - this does not add appreciably to the run-time, since generating proposed states can be done efficiently and the computational effort is dominated by calculating p_{accept}. In this case, the acceptance probability is

$$\begin{aligned} p_{\text{accept}} &= \min\left(1, \frac{P(\vec{n}_i, \vec{\alpha}_i|N)Q(\vec{n}_{i-1}, \vec{\alpha}_{i-1}|N)}{P(\vec{n}_{i-1}, \vec{\alpha}_{i-1}|N)Q(\vec{n}_i, \vec{\alpha}_i|N)}\right) \\ &= \min\left(1, \frac{P(\vec{n}_i, \vec{\alpha}_i, N)Q(\vec{n}_{i-1}, \vec{\alpha}_{i-1}, N)}{P(\vec{n}_{i-1}, \vec{\alpha}_{i-1}, N)Q(\vec{n}_i, \vec{\alpha}_i, N)}\right) \\ &= \min\left(1, \frac{P(\vec{n}_i, \vec{\alpha}_i)Q(\vec{n}_{i-1}, \vec{\alpha}_{i-1})}{P(\vec{n}_{i-1}, \vec{\alpha}_{i-1})Q(\vec{n}_i, \vec{\alpha}_i)}\right), \end{aligned} \tag{S14}$$

where in the second line we used the definition of conditional probability $P(\vec{n}_i, \vec{\alpha}_i, N) = P(\vec{n}_i, \vec{\alpha}_i|N)P(N)$, and the $P(N)$'s cancel and so do the $Q(N)$'s. In the third line, we know that if we are post-selecting, all $\vec{n}$ will automatically satisfy N so it is a redundant variable. Hence an identical p_{accept} can be used when fixing N.

We outline the algorithm below. To sample from a state with vector of means $\boldsymbol{R}$ and covariance matrix $\boldsymbol{V}$:

1. Use the Williamson decomposition to write $\boldsymbol{V} = \boldsymbol{T} + \boldsymbol{W}$, where $\boldsymbol{T}$ is the covariance matrix of a pure state. Calculate the matrix $\boldsymbol{B}$ based on $\boldsymbol{T}$.
2. Sample a displacement vector $\boldsymbol{R'}$ from the multivariate normal distribution $\boldsymbol{R'} \sim \mathcal{N}(\boldsymbol{R}, \boldsymbol{W})$. Calculate the complex displacement $\vec{\alpha}'_1$ from $\boldsymbol{R'}$.
3. Sample a photon pattern $\vec{n}_1$ from $Q(\vec{n}|\boldsymbol{B}, \vec{\alpha}_1)$. This involves sampling from Poissonian distributions.
4. Start a Markov chain from the state $(\vec{n}_1, \vec{\alpha}'_1)$.
5. For step i in the Markov chain from 2 to the desired length:
   (a) Sample a new displacement vector $\vec{\alpha}'_i$.
   (b) Sample a new photon pattern $\vec{n}_i$ from $Q(\vec{n})$ for the pure state with displacement $\vec{\alpha}'_i$ and covariance matrix $\boldsymbol{T}$.
   (c) Calculate the acceptance probability p_{accept}.
   (d) Add $(\vec{n}_i, \vec{\alpha}'_i)$ to the chain with probability p_{accept}, otherwise add the previous state again.
6. Keep only the $\vec{n}$ values in the chain (ignore $\vec{\alpha}'$). Discard the first τ_{burn} samples and then keep every 1 in τ_{thin} samples.

C. Threshold detector GBS

For MIS with threshold detectors, we also need to include the fan out of each mode into sub-detectors, where we only register the position of the 'first' photon. So we now expand the sample space to include a variable describing this position, x. We take the limit of a large number of sub-detectors where x becomes a continuous variable, and choose larger x to correspond to 'earlier' detections.

The POVM element for a click outcome where the first photon is at position x can be written:

$$\pi_c(x) = \sum_{n=1}^{\infty} p(x|n) |n\rangle \langle n| , \tag{S15}$$

with $p(x|n) = nx^{n-1}$. $\pi_c(x)$ is closely related to the POVM element for measuring a single photon after a loss of x:

$$\Pi_L(x) = \sum_{j=1}^{\infty} j(1-x)x^{j-1} |j\rangle \langle j| = (1-x)\pi_c(x). \tag{S16}$$

Hence we can express the probability of a click pattern $\vec{c}$ with an accompanying $\vec{x}$ in terms of the probability of obtaining the same pattern of single photons, but from a covariance matrix $\boldsymbol{V}(\vec{x})$ where the loss x_m has been applied to the mth mode (for unoccupied modes $x_m = 0$):

$$P_c(\vec{c}, \vec{x}, \vec{\alpha}') = \frac{P(\vec{\alpha}')P_n(\vec{c}|\vec{x}, \vec{\alpha}')}{\prod_m (1-x_m)}, \tag{S17}$$

where as before $\vec{\alpha}'$ is a complex displacement vector chosen from a multivariate normal distribution. Since $\boldsymbol{V}(\vec{x})$ is a mixed state, we expand it as an ensemble of pure states with differing displacement vectors $\vec{\alpha}''$:

$$P_c(\vec{c}, \vec{x}, \vec{\alpha}', \vec{\alpha}'') = \frac{P(\vec{\alpha}')P(\vec{\alpha}''|\vec{x}, \vec{\alpha}')P_n(\vec{c}|\vec{x}, \vec{\alpha}'')}{\prod_m (1-x_m)}, \tag{S18}$$

where $P(\vec{\alpha}''|\vec{x}, \vec{\alpha}')$ is the probability distribution of $\vec{\alpha}''$, depending on the applied loss, $\vec{x}$, and the complex displacement before the loss, $\vec{\alpha}'$. $P_n(\vec{c}|\vec{x}, \vec{\alpha}'')$ is the photon number pattern probability of a pure state and can be calculated with an $N_c \times N_c$ loop hafnian, in time $\mathcal{O}(N_c^3 2^{N_c/2})$, resulting in a quadratic speedup compared to a Torontonian. If we sample $(\vec{c}, \vec{x}, \vec{\alpha}'', \vec{\alpha}')$ and then ignore the $\vec{x}$ and $\vec{\alpha}$ outcomes, this is equivalent to sampling from $P_c(\vec{c})$ as desired.

To generate proposal samples, we begin by generating a displacement vector $\vec{\alpha}'$ and photon number pattern $\vec{n}$ as in Appendix 3 B. Then a $\vec{x}$ vector can be generated by sampling from $p(x|n)$ for each element, and a click pattern $\vec{c}$ taken by reducing each > 0 element of $\vec{n}$ to a 1. The loss $\vec{x}$ is applied to the state, resulting in an updated displacement $\vec{\alpha}'(x)$ and covariance matrix $\boldsymbol{V}(\vec{x})$, from which a Williamson decomposition can be used to sample a pure state - with a displacement $\vec{\alpha}''$ and covariance matrix T'.

The proposal probability, marginalised over $\vec{n}$, can be written

$$Q_c(\vec{c}, \vec{x}, \vec{\alpha}', \vec{\alpha}'') = P(\vec{\alpha})P(\vec{\alpha}''|\vec{x}, \vec{\alpha}')Q_c(\vec{c}, \vec{x}|\vec{\alpha}'), \tag{S19}$$

where we note that the proposal distribution for ($\vec{c}$, $\vec{x}$) is conditioned on $\vec{\alpha}'$ rather than $\vec{\alpha}''$, which is the last variable to be chosen. As with the target distribution, this probability can be rewritten in terms of a pattern of single photons after application of a loss:

$$Q_c(\vec{c}, \vec{x}|\vec{\alpha}') = \frac{Q_n(\vec{c}|\vec{x}, \vec{\alpha}')}{\prod_m (1 - x_m)}. \tag{S20}$$

The probability of detecting a pattern of single photons from IPS after loss is still given by the loop hafnian of a non-negative matrix:

$$Q_n(\vec{c}|\vec{x}, \vec{\alpha}') \propto \text{lhaf}(\boldsymbol{C}_{\vec{c}}(\vec{x})), \tag{S21}$$

where we take

$$\boldsymbol{C}_{j,k}(\vec{x}) = (1 - x_j)(1 - x_k)|B_{j,k}|^2, \tag{S22}$$

except for diagonal elements

$$\boldsymbol{C}_{j,j}(\vec{x}) = (1 - x_j)\left(|\alpha_j|^2 + \sum_k x_k |B_{j,k}|^2\right), \tag{S23}$$

and form $\boldsymbol{C}_{\vec{c}}(\vec{x})$ by keeping the elements of $\boldsymbol{C}$ where $c = 1$. This results in an acceptance probability:

$$p_{\text{accept}} = \min\left(1, \frac{P_n(\vec{c}_i|\vec{x}_i, \vec{\alpha}_i'')Q_n(\vec{c}_{i-1}|\vec{x}_{i-1}, \vec{\alpha}_{i-1}')}{Q_n(\vec{c}_i|\vec{x}_i, \vec{\alpha}_i')P_n(\vec{c}_{i-1}|\vec{x}_{i-1}, \vec{\alpha}_{i-1}'')}\right). \tag{S24}$$

We outline the steps of the MIS algorithm below.

1. Use the Williamson decomposition to write $\boldsymbol{V} = \boldsymbol{T} + \boldsymbol{W}$, where $\boldsymbol{T}$ is the covariance matrix of a pure state.
2. Sample the starting state from the proposal distribution
   (a) Sample a complex displacement vector $\vec{\alpha}_1'$.
   (b) Sample a photon pattern $\vec{n}_1$ from $Q(\vec{n}|\vec{\alpha}')$. Find $\vec{c}_1$ from $\vec{n}_1$ by fixing all $n_i > 1$ as $c_i = 1$.
   (c) If post-selecting on the number of clicks, repeat the above steps until $\vec{c}$ contains the desired number of clicks.
   (d) Sample the loss, $\vec{x}_1$, conditional on the photon number pattern $\vec{n}_1$ using $p(x|n)$.
   (e) Apply the loss $\vec{x}_1$ to the displacement vector $\vec{\alpha}_1'$ and the covariance matrix $\boldsymbol{T}$, resulting in $\vec{\alpha}_1'(\vec{x})$ and $\boldsymbol{V}(\vec{x})$.
   (f) Perform a Williamson decomposition on the mixed state to obtain a pure state covariance matrix $\boldsymbol{T'}$ and sample a new complex displacement vector $\vec{\alpha}_1''$.
3. Start the Markov chain from the state $(\vec{c}_1, \vec{x}_1, \vec{\alpha}_1', \vec{\alpha}_1'')$. Calculate the target probability using equation S18 and the proposal probability using equation S19.
4. For step i in the Markov chain from 2 to the desired length:
   (a) Sample another proposal sample $(\vec{c}_i, \vec{x}_i, \vec{\alpha}_i', \vec{\alpha}_i'')$.
   (b) Calculate the target and proposal probabilities for this state.
   (c) Calculate the acceptance probability p_{accept} using equation S24.
   (d) Add $(\vec{c}_i, \vec{x}_i, \vec{\alpha}_i', \vec{\alpha}_i'')$ to the chain with probability p_{accept}, otherwise add the previous state again.
5. Keep only the $\vec{c}$ values in the chain (ignore $\vec{x}$, $\vec{\alpha}'$ and $\vec{\alpha}''$). Discard the first τ_{burn} samples and then keep every 1 in τ_{thin} samples.

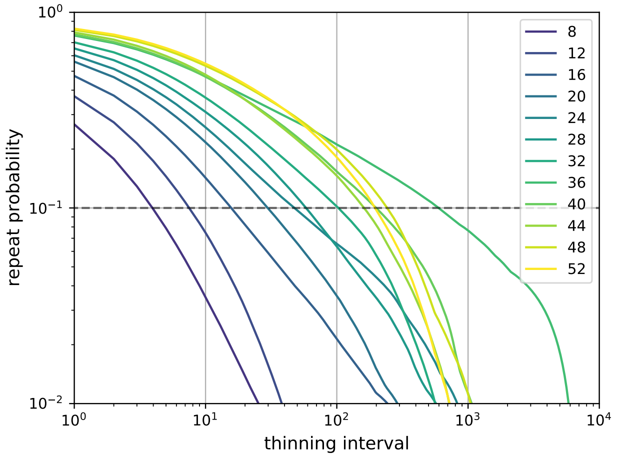

Supplementary Figure S4. **Estimated probability of repeated samples.** Calculated for each M from 10 Haar random unitary matrices, with a 10,000 long MIS chain for each unitary.

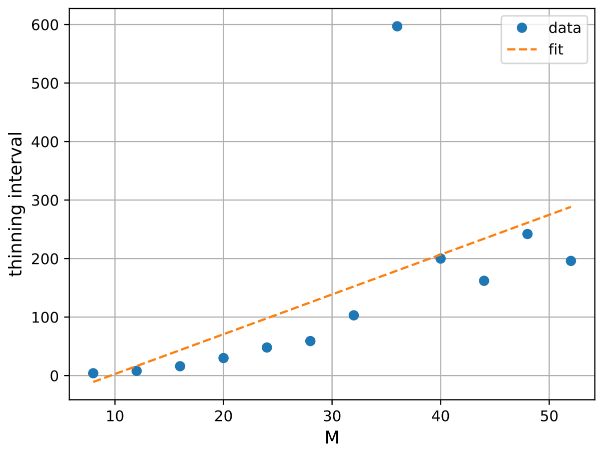

Supplementary Figure S5. **Thinning interval scaling.** Thinning intervals generated from the data in Fig. **S4**, by choosing 0.1 as an acceptable repeat probability. The $M = 36$ data point is far from the line of best fit, likely due to a sample with an anomalously large separation between target and proposal probabilities. The linear fit follows $\tau_{thin} = 6.81M - 65.3$.

D. Thinning interval and burn-in time scaling

To investigate the scaling of our algorithms, we fix the number of photons to the mean photon number number, rounded to the nearest integer. The tests described in this section are applicable to both PNRD and threshold GBS unless stated otherwise. However, we only implement them for the number resolving case.

It is important to be able to predict the run-time of simulations before they are performed. For MIS methods, this is challenging as thinning intervals and burn-in times depend on how close the proposal distribution is to the target distribution. To construct heuristics to allow us to make these predictions, we investigate how the thinning interval and burn-in time scale with the number of modes M. However, we wish to highlight that the requirements on accuracy and sample autocorrelation will vary depending on what is desired from the simulation. Therefore the results in this section should be viewed as a guide for how to predict the scaling, rather than as a prescriptive guide for what parameters should be used.

To predict the thinning interval, τ_{thin}, we investigate systems of different sizes with M varied between 8 and 52 in steps of 4. For each M we choose 10 Haar random interferometers, and implement an MIS chain with 10,000 steps. In Fig. **S4**, we plot the estimated probability of a sample being repeated as a function of the thinning interval. From

this we extract the thinning interval required to suppress the repeat probability to 0.1 for each M and perform a linear fit on this data, shown in Fig. **S5**.

The data for $M = 36$ appears anomalous. We believe this is caused by one of the chains drawing a proposal sample which has an unusually large target/proposal probability ratio. Such events can cause chains to reject a very large number of samples before accepting a new proposal sample. The large degree of autocorrelation which is created by events such as this are an intrinsic drawback of the MIS method, and so we do not discard this data.

The second parameter we need to determine is the burn-in time, τ_{burn}. We know that the chain begins sampling from the proposal distribution and over time converges to the target distribution. It will converge continuously, getting asymptotically closer to the target distribution, but at some point it will be close enough that the change will not be noticeable from finite sample sizes. Therefore, we can analyse when our distribution appears to be stationary. We provide two tests to predict how the burn-in time scales with the number of modes.

For the first, we use a Bayesian likelihood ratio test for each burn-in time until we see no improvement. The likelihood ratio tests whether a set of samples s is more likely to have come from the target distribution or an adversary distribution. To test how close our distribution is to the target, $\mathcal{P}$, or proposal, $\mathcal{Q}$, we choose the proposal distribution as the adversary. We begin with the ratio

$$\chi = \frac{p(\mathcal{P}|s)}{p(\mathcal{Q}|s)} = \frac{p(s|\mathcal{P})p(\mathcal{P})p(s)}{p(s|\mathcal{Q})p(\mathcal{Q})p(s)}. \tag{S25}$$

If we assume equal priors $p(\mathcal{P}) = p(\mathcal{Q})$, this simplifies to

$$\chi = \frac{p(s|\mathcal{P})}{p(s|\mathcal{Q})}. \tag{S26}$$

Assuming that the probability distribution is either $\mathcal{P}$ or $\mathcal{Q}$ and so $p(\mathcal{P}|s) + p(\mathcal{Q}|s) = 1$, we can write

$$p(\mathcal{P}|s) = p(\mathcal{Q}|s)\chi = (1 - p(\mathcal{P}|s))\chi \tag{S27}$$

$$\implies p(\mathcal{P}|s) = \frac{\chi}{1+\chi}. \tag{S28}$$

Here our samples s_i are described by $(\vec{\alpha}_i, \vec{n}_i)$ which we sample from the chain. So we can write $p(s_i|\mathcal{P}) = p(\vec{\alpha}_i, \vec{n}_i|\mathcal{P}) = p(\vec{\alpha}_i|\mathcal{P})p(\vec{n}_i|\vec{\alpha}_i, \mathcal{P})$. For purposes of benchmarking the efficiency, we fix the number of photons and so we have to adjust for post-selecting on N photons. We still assume equal priors, now $p(\mathcal{P}|N) = p(\mathcal{Q}|N)$. So the likelihood ratio becomes

$$\chi = \frac{p(s|\mathcal{P}, N)}{p(s|\mathcal{Q}, N)} = \prod_i \frac{P(\vec{\alpha}_i, \vec{n}_i|N)}{Q(\vec{\alpha}_i, \vec{n}_i|N)} \tag{S29}$$

$$= \prod_i \frac{P(\vec{n}_i, N|\vec{\alpha}_i)Q(N)}{Q(\vec{n}_i, N|\vec{\alpha}_i)P(N)}, \tag{S30}$$

where we use the fact that $P(\vec{\alpha}_i) = Q(\vec{\alpha}_i)$. This only requires the calculation of pure-state probabilities and the probability of getting N photons in both the proposal and target distribution. This can be done for PNRDs with no additional cost to the sampling algorithm as we must calculate the pure-state probabilities in the formation of our chain, and calculating the probabilities of N photons is efficient. However, for threshold detectors, although we could add the x variable, we are not aware of a way to calculate the probability of N_c clicks for either distribution. Therefore we do not apply this test to threshold detectors.

We evaluate this probability for all burn-in times up to 100, for an increasing sample size up to 100. As we increase the sample size, the likelihood should eventually converge to either 0 (if it fails) or 1 (if it passes) - see Fig. **S6**. The closer the sampled distribution is to the target, the faster it converges to 1, so we can test how close our distribution is for different burn-in times by sampling and comparing the rate of the convergence to 1. To isolate the burn-in for testing, we need to start a new chain every time we sample. As a metric for comparing the rates of convergence, we find the sample size required to reach a likelihood of 0.95. As we increase the burn-in time, the sample size should decrease and approach the minimum. We find the burn-in time at which the sample size is within 5% of the estimated minimum. Each likelihood is estimated by averaging over 1000 Haar random unitaries. Despite this, the likelihood still gives quite noisy data and we further average over a range of 10 burn-in times, ie the likelihood at burn-in i is given by the average of the likelihood for burn-in times between i and $i + 9$ (see Fig. **S7**). The minimum is estimated in a similar way where we average over the last 20 burn-in times, when we can assume it has converged. Fig. **S8** shows

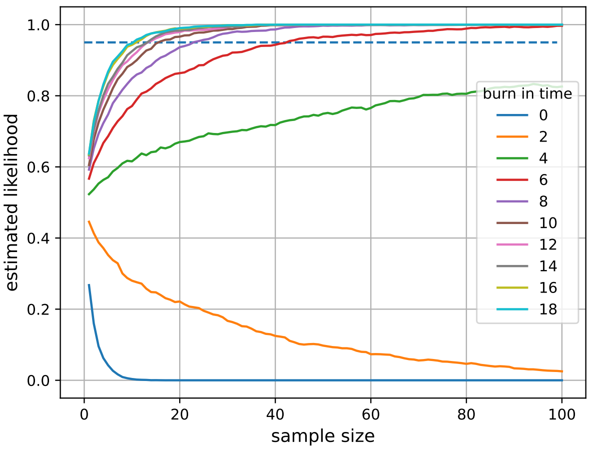

Supplementary Figure S6. **Likelihood based burn-in time benchmarking.** The estimated likelihood ratio as a function of the number of samples included for a range of burn-in times. The plot shows how the likelihood converges to either 0 if the test fails or 1 if it passes for increasing burn-in times averaged over 1000 Haar random unitaries in 28 modes. We wish to find the sample size at which the likelihood ratio reaches 0.95 as indicated by the dashed line.

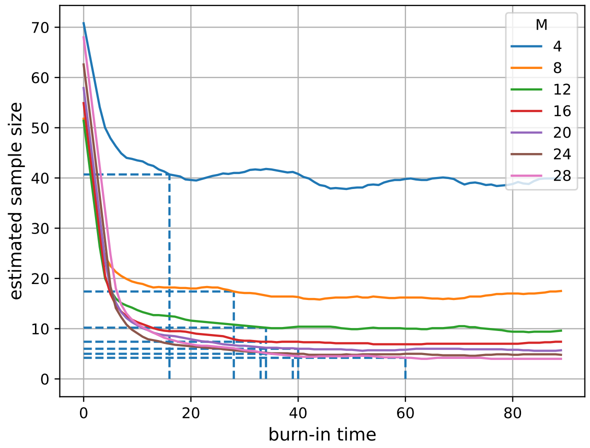

Supplementary Figure S7. **Likelihood based burn-in time convergence.** The estimated sample size required to give a likelihood ratio of 0.95 for burn-in times between 0 and 100 (averaging over 10 burn-in times). We wish to find the burn-in time beyond which we see no improvement in the number of samples required, as indicated by the dashed lines.

the estimated burn-in time for up to 28 modes and we extrapolate the linear fit to give an estimate of $\tau_{\text{burn}} = 155$ for 100 modes.

For the second test, we note that the rate of accepting a proposed sample decreases towards an asymptotic minimum value as the chain converges. This minimum value would be reached only when sampling from the target distribution. We estimate the probability of accepting at each burn-in time up to 300 by running 10,000 chains and counting the number of times we accept for a Haar random unitary. As with the likelihood test, we still have noisy data and smooth out the curve by averaging across 10 burn-in times. We choose the burn-in time at which the probability of accepting is no more than 0.001 greater than the estimated minimum value. Again we estimate the minimum value from the end of our chain, averaging over the last 50 burn-in times. As long as the estimated burn-in time is significantly before the end of the chain, we can be reassured that the probability of accepting is changing slowly enough to consider the chain to have converged by the maximum burn-in time we test. See Fig. **S9** for an example of how the acceptance rate varies with the chain length. We run this test for 10 Haar random unitaries and find the average burn-in time for each M up to 24, shown in Fig. **S10**, which extrapolating gives an estimate of $\tau_{\text{burn}} = 785$ for 100 modes.

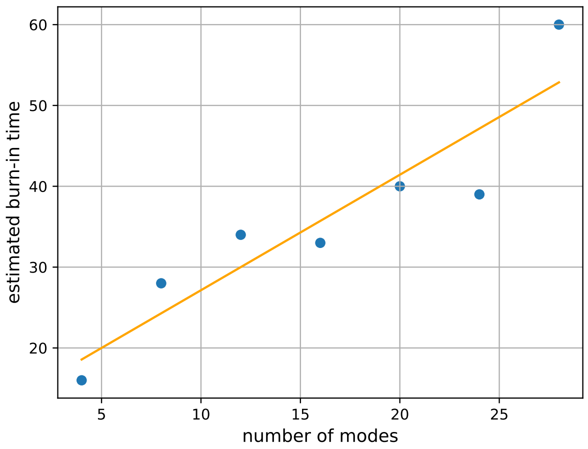

Supplementary Figure S8. **Likelihood based burn-in time scaling.** The estimated burn-in time from the likelihood test as a function of the number of modes. The likelihood is calculated for increasing sample size for up to a burn-in time of 100. We estimate at which burn-in time we do not see an improvement to the likelihood. We find the sample size required to reach a likelihood of 0.95 and find the burn-in at which it is within 5% of the final value. Each likelihood was estimated by averaging over 1000 Haar random unitaries. We use a linear fit of the data to predict a relationship of $\tau_{\text{burn}} = 1.43M + 12.86$.

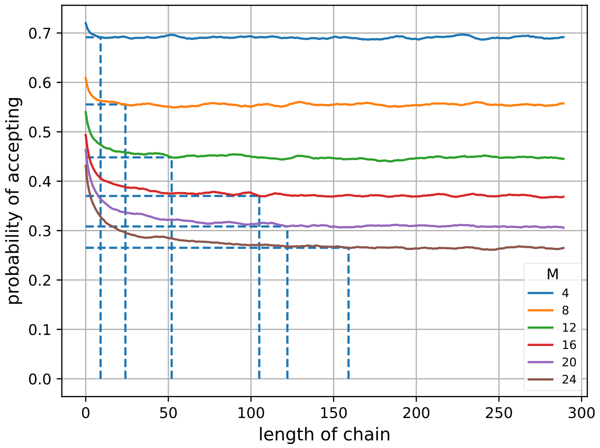

Supplementary Figure S9. **Acceptance rate based burn in time convergence.** The acceptance rate as a function of the length of the chain for various values of M, the number of modes. We find the point in the chain at which the acceptance rate becomes approximately constant. This plot shows an example for one Haar random unitary for each M. The dashed horizontal lines show when the curve reaches withing 0.001 of the estimated final value.

We note that these two tests give significantly different estimates for the burn-in times. This is likely due to two reasons. The first is that the likelihood may be less sensitive to the convergence and doesn't distinguish between two close distributions as well. The data for this test is more noisy and so that may hide small differences in the distributions. The second is that we fix it to have converged further for the acceptance rate test. It is an arbitrary choice to decide how close you require the distribution to be to the target distribution. In both tests, we are limited by how noisy our data is from finite sampling. If our acceptance rate test is less noisy, we are able to find the burn-in times for a better convergence. The important findings from our numerical analysis are that the burn-in time seems to scale approximately linearly with the number of modes and the gradient of the scaling depends on how close you want the distribution you are sampling from to be to the target distribution.

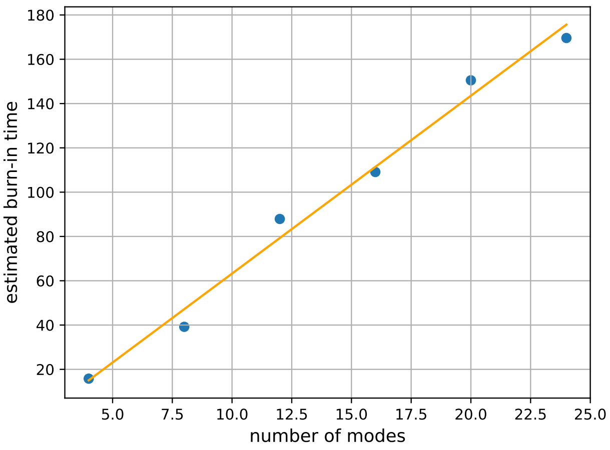

Supplementary Figure S10. **Acceptance rate based burn in time scaling.** The estimated burn-in time from the acceptance rate test as a function of the number of modes. We estimate the burn-in beyond which the probability of accepting the proposed sample is approximately constant. Each burn-in was estimated by averaging over 10 Haar random unitaries. We use a linear fit of the data to predict a relationship of $\tau_{\mathrm{burn}} = 8.03M - 17.06$.

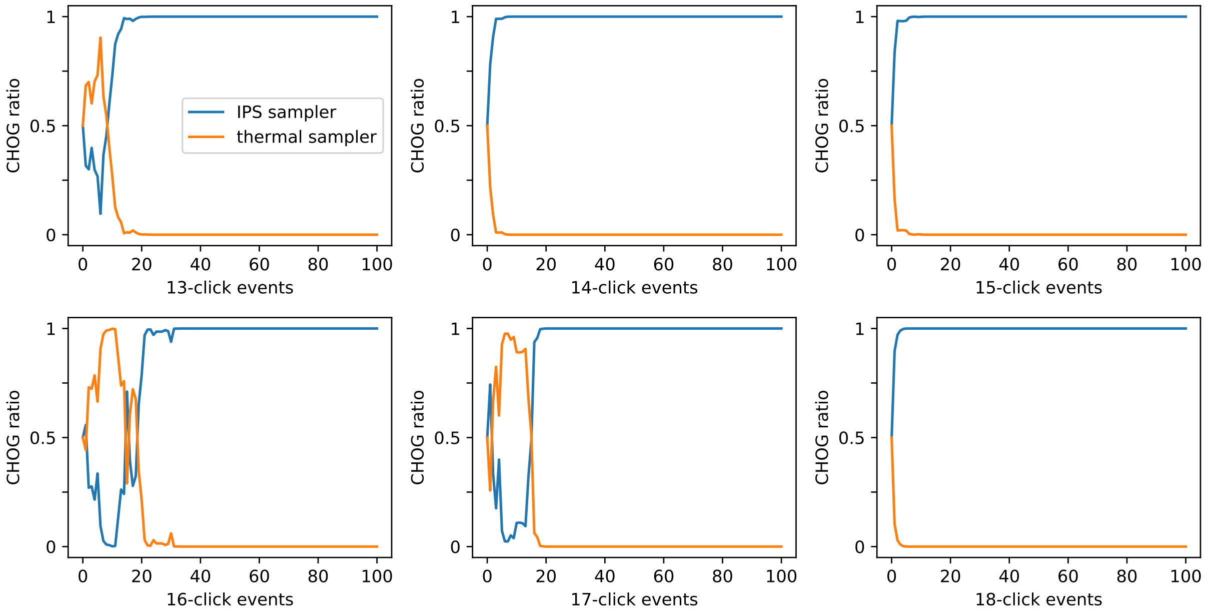

Supplementary Figure S11. **CHOG ratio validation of IPS against thermal sampler.** $M = 52$ CHOG tests for different total click numbers to determine the relative likelihood of IPS and thermal samples from the ideal click distribution. Convergence to 1 for the IPS samplers indicates they are more likely to have come from the click distribution than the thermal samples.

4. VALIDATION TESTS

A. CHOG ratio

As we approach a scale where exact validation of samples becomes unfeasible, we can use the Chen Heavy Output Generation (CHOG) ratio test outlined in ref. [6]. Certain output patterns from a random optical network occur more frequently due to constructive interference and it is thought to be difficult to replicate this observation with classical samplers. This adversarial test assesses the relative likelihood of two sets of samples ('trial' and 'adversarial') being

drawn from a given ideal distribution. As samples are drawn, the CHOG ratio is updated:

$$r_{\text{CHOG}} = \frac{P_{\text{ideal}}(\text{samples}_{\text{trial}})}{P_{\text{ideal}}(\text{samples}_{\text{trial}}) + P_{\text{ideal}}(\text{samples}_{\text{adv}})} \tag{S31}$$

$$= \left(1 + \prod_j \frac{P_{\text{ideal}}(\text{sample}_{\text{adv}}(j))}{P_{\text{ideal}}(\text{sample}_{\text{trial}}(j))}\right)^{-1}. \tag{S32}$$

Convergence to a value of 1 indicates that the trial samples were more likely to be drawn from the ideal distribution than the adversarial samples. In the case of click detection samples, the probabilities of observing these samples from the ideal (squeezed) distribution are calculated using the Torontonian.

In the work by USTC [6], click samples are drawn from the trial GBS experiment and an adversarial thermal sampler. A value of 1 indicates that the GBS samples were more likely to be drawn from the ideal distribution than the thermal samples. For Jiǔzhāng, GBS samples corresponding to fixed total numbers of clicks of between 26 and 38 were validated against a thermal sampler.

1. *IPS vs thermal*

Our IPS sampler (used as a proposal distribution for the MIS algorithm) naturally incorporates constructive interference of pairs of photons. Here, we use it to generate trial samples and apply the CHOG test to validate against an adversarial thermal sampler. For the IPS sampler we use a covariance matrix corresponding to 13 sources of two-mode squeezed vacuum, with squeezing parameter $r = 1.55$ and transmission $\eta = 0.3$, injected into a Haar random 52-mode unitary interferometer. The covariance matrix for the thermal sampler is constructed using the same transmissions and unitary interferometer, but now injected with 26 thermal states, each with mean photon number $n_{th} = \sinh^2(r)$. We update the CHOG ratio using equation S32 and results for click numbers of between 13 and 18 are shown in Fig. **S11**. The convergence to 1 for the IPS sampler shows that those samples are more likely to have been drawn from the ideal distribution than the thermal samples.

Hence, we have shown that our IPS sampler – from which samples can be efficiently drawn classically – passes the CHOG test against a thermal sampler in a similar way to the experimental GBS samples from Jiǔzhāng. This challenges the usefulness of the CHOG test against a thermal adversarial sampler in validating quantum computational complexity of GBS. It also suggests that the IPS distribution should be used as an adversary model to test against in future experiments. Because the IPS distribution contains no interference between different photon pairs, it could be considered as the distribution generated by squeezers with zero spectral purity. Following this intuition, we also suggest a finite purity adversary (i.e. squeezing across ≥ 2 Schmidt modes) as another important, more challenging, model to test against. Calculating probabilities of Gaussian states in the presence of spectral impurity has been investigated in ref. [25].

2. *MIS vs IPS*

Our MIS method takes the IPS as its proposal distribution and should then converge to the target (Torontonian) distribution. Here, we use a CHOG ratio test to validate trial MIS samples against the IPS distribution as the adversary. Our discussion in the previous section showed that IPS samples are more likely to be drawn from the ideal distribution than thermal samples, and so here they should provide a more stringent test.

We use a covariance matrix corresponding to 6 sources of two-mode squeezed vacuum, with squeezing parameter $r = 1.55$ and transmission $\eta = 0.3$, injected into a Haar random 24-mode unitary interferometer. We draw 10^5 samples from the IPS distribution and use these in an MIS chain with burn-in of 50 and a thinning interval of 10. We then post-select for samples of different fixed total click numbers and update the CHOG ratio. Results for click numbers of between 9 and 12 are shown in Fig. **S12**. The convergence to 1 for the MIS samples shows that they are more likely to have been drawn from the ideal distribution than the starting IPS samples, and this is indicative of convergence of the chain.

B. Output correlations

In this section we compare statistical correlations in output click patterns obtained from IPS samples to the exact, Torontonian distribution. We use a covariance matrix corresponding to 8 sources of two-mode squeezed vacuum, with

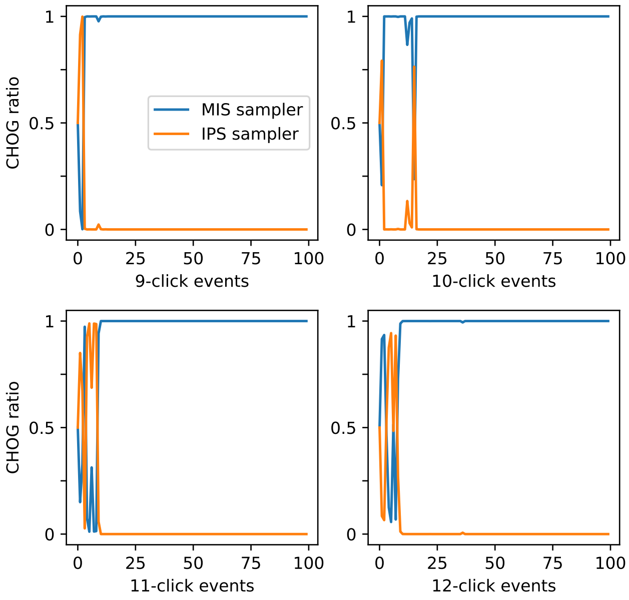

Supplementary Figure S12. **CHOG ratio validation of MIS against IPS.** $M = 24$ CHOG tests for different total click numbers to determine the relative likelihood of MIS and IPS samples from the ideal click distribution. Convergence to 1 for the MIS samplers indicates they are more likely to have come from the click distribution than the IPS samples.

squeezing parameter $r = 1.55$ and transmission $\eta = 0.3$, injected into a Haar random 32-mode unitary interferometer. We draw 10^6 IPS samples over the 32 modes and convert them to click patterns. These can be used to estimate click probabilities over different output modes. We also use the Torontonian to calculate exact click probabilities.

We estimate the single-mode click probabilities from IPS samples by dividing the number of clicks on a specific mode in those samples by the total. These are compared to the exact probabilities in Fig. **S13**a. We find that the values estimated from IPS samples agree with the ideal.

Next we consider second-order correlators that have been proposed as a benchmark for GBS [49]. For some state of light emerging from an optical network, they are defined as:

$$C_{i,j} = \langle \Pi_i \Pi_j \rangle - \langle \Pi_i \rangle \langle \Pi_j \rangle, \tag{S33}$$

where the projector $\Pi_i = \mathbb{I} - |0\rangle_i \langle 0|_i$ corresponds to a click on mode i. We use IPS samples to estimate the probability of clicks on modes i and j and, together with the single-mode click probabilities from earlier, estimate $C_{i,j}$ for all pairs of output modes. A comparison to exact values is given in Fig. **S13**b. Although the points do not fall on a line of unit gradient, the positive correlation between the values indicates that the IPS samples are capturing underlying second-order correlations in the state of light from a GBS device.

Following [30], we also investigate higher third-order correlations of our IPS samples. These are defined as:

$$C_{i,j,k} = \langle \Pi_i \Pi_j \Pi_k \rangle - \langle \Pi_i \Pi_j \rangle \langle \Pi_k \rangle - \langle \Pi_i \Pi_k \rangle \langle \Pi_j \rangle - \langle \Pi_j \Pi_k \rangle \langle \Pi_i \rangle + 2 \langle \Pi_i \rangle \langle \Pi_j \rangle \langle \Pi_k \rangle. \tag{S34}$$

The estimated values are compared to the exact in Fig. **S13**c. Again a positive correlation in the plot of estimated against exact values suggests IPS samples encode some of the higher-order correlations present in the GBS statistics.

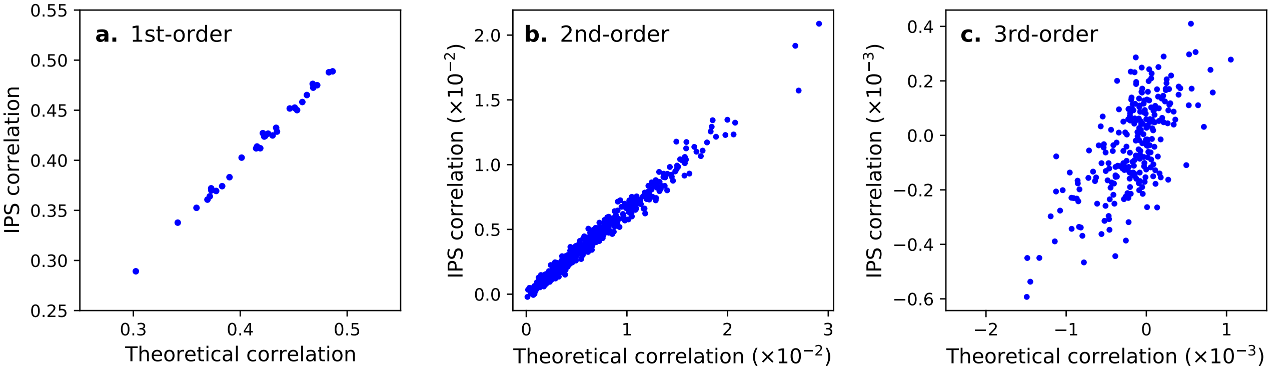

Supplementary Figure S13. **Correlation function benchmarking of IPS distribution.** (a)-(c) First-, second-, and third-order correlation functions for an $M = 32$ GBS task (see equations S33 and S34 in the main text for definitions). Theoretical values are calculated using Torontonians and values for the IPS distribution are estimated using 10^6 samples. In (b) we plot all $\binom{32}{2}$ second-order correlator values, while in (c) we plot only a subset of all possible three-mode correlations.

REFERENCES AND NOTES

1. S. Aaronson, A. Arkhipov, The computational complexity of linear optics, in *Proceedings of the Forty-Third Annual ACM Symposium on Theory of Computing* (ACM, 2011), pp. 333–342.

2. A. P. Lund, A. Laing, S. Rahimi-Keshari, T. Rudolph, J. L. O'Brien, T. C. Ralph, Boson sampling from a Gaussian state. *Phys. Rev. Lett.* **113**, 100502 (2014).

3. C. S. Hamilton, R. Kruse, L. Sansoni, S. Barkhofen, C. Silberhorn, I. Jex, Gaussian boson sampling. *Phys. Rev. Lett.* **119**, 170501 (2017).

4. R. Kruse, C. S. Hamilton, L. Sansoni, S. Barkhofen, C. Silberhorn, I. Jex, Detailed study of Gaussian boson sampling. *Phys. Rev. A* **100**, 032326 (2019).

5. N. Quesada, Franck-Condon factors by counting perfect matchings of graphs with loops. *J. Chem. Phys.* **150**, 164113 (2019).

6. H.-S. Zhong, H. Wang, Y.-H. Deng, M.-C. Chen, L.-C. Peng, Y.-H. Luo, J. Qin, D. Wu, X. Ding, Y. Hu, P. Hu, X.-Y. Yang, W.-J. Zhang, H. Li, Y. Li, X. Jiang, L. Gan, G. Yang, L. You, Z. Wang, L. Li, N.-L. Liu, C.-Y. Lu, J.-W. Pan, Quantum computational advantage using photons. *Science* **370**, 1460–1463 (2020).

7. N. Quesada, J. M. Arrazola, N. Killoran, Gaussian boson sampling using threshold detectors. *Phys. Rev. A* **98**, 062322 (2018).

8. D. Grier, D. J. Brod, J. M. Arrazola, M. B. de Andre Alonso, N. Quesada, The complexity of bipartite Gaussian boson sampling. arXiv:2110.06964 [quant-ph] (13 October 2021).

9. N. Quesada, R. S. Chadwick, B. A. Bell, J. M. Arrazola, T. Vincent, H. Qi, R. García-Patrón, Quadratic speedup for simulating Gaussian boson sampling. arXiv:2010.15595 [quant-ph] (29 October 2020).

10. A. Neville, C. Sparrow, R. Clifford, E. Johnston, P. M. Birchall, A. Montanaro, A. Laing, Classical boson sampling algorithms with superior performance to nearterm experiments. *Nat. Phys.* **13**, 1153–1157 (2017).

11. N. Quesada, J. M. Arrazola, Exact simulation of Gaussian boson sampling in polynomial space and exponential time. *Phys. Rev. Res.* **2**, 023005 (2020).

12. B. Wu, B. Cheng, F. Jia, J. Zhang, M.-H. Yung, X. Sun, Speedup in classical simulation of Gaussian boson sampling. *Sci. Bull.* **65**, 832–841 (2020).

13. B. Gupt, J. M. Arrazola, N. Quesada, T. R. Bromley, Classical benchmarking of Gaussian Boson Sampling on the Titan supercomputer. *Quantum Inf. Process* **19**, 249 (2020).

14. A. Serafini, *Quantum Continuous Variables* (CRC Press, 2017).

15. H. J. Ryser, *Combinatorial Mathematics* (Mathematical Association of America, 1963), vol. 14.

16. V. S. Shchesnovich, Asymptotic evaluation of bosonic probability amplitudes in linear unitary networks in the case of large number of bosons. *Int. J. Quantum Inf.* **11**, 1350045 (2013).

17. M. Tichy, "Entanglement and interference of identical particles," thesis, Albert-Ludwigs-Universität Freiburg, Freiburg (2011).

18. S. Chin, J. Huh, Generalized concurrence in boson sampling. *Sci. Rep.* **8**, 6101 (2018).

19. R. Kan, From moments of sum to moments of product. *J. Multivar. Anal.* **99**, 542–554 (2008).

20. A. Björklund, B. Gupt, N. Quesada, A faster hafnian formula for complex matrices and its benchmarking on a supercomputer. *J. Exp. Algorithmics* **24**, 1–17 (2019).

21. M. Cygan, M. Pilipczuk, Faster exponential-time algorithms in graphs of bounded average degree. *Inf. Comput.* **243**, 75–85 (2015).

22. K. Balasubramanian, "Combinatorics and diagonals of matrices," thesis, Indian Statistical Institute, Calcutta (1980).

23. E. Bax, J. Franklin, A finite-difference sieve to count paths and cycles by length. *Inf. Process. Lett.* **60**, 171–176 (1996).

24. D. G. Glynn, The permanent of a square matrix. *Eur. J. Comb.* **31**, 1887–1891 (2010).

25. O. F. Thomas, W. McCutcheon, D. P. S. McCutcheon, A general framework for multimode Gaussian quantum optics and photo-detection: Application to Hong–Ou–Mandel interference with filtered heralded single photon sources. *APL Photonics* **6**, 040801 (2021).

26. J. S. Liu, Metropolized independent sampling with comparisons to rejection sampling and importance sampling. *Stat. Comput.* **6**, 113–119 (1996).

27. J. S. Liu, *Monte Carlo Strategies in Scientific Computing* (Springer Publishing Company Incorporated, 2008).

28. L. Devroye, Nonuniform random variate generation, in *Handbooks in Operations Research and Management Science*, S. G. Henderson, B. L. Nelson, Eds. (Elsevier, 2006), vol. 13, pp. 83–122.

29. F. Mezzadri, How to generate random matrices from the classical compact groups. *Not. Am. Math. Soc* **54**, 592–604 (2006).

30. H.-S. Zhong, Y.-H. Deng, J. Qin, H. Wang, M.-C. Chen, L.-C. Peng, Y.-H. Luo, D. Wu, S.-Q. Gong, H. Su, Y. Hu, P. Hu, X.-Y. Yang, W.-J. Zhang, H. Li, Y. Li, X. Jiang, L. Gan, G. Yang, L. You, Z.

Wang, L. Li, N.-L. Liu, J.-J. Renema, C.-Y. Lu, J.-W. Pan, Phase-programmable Gaussian boson sampling using stimulated squeezed light. *Phys. Rev. Lett.* **127**, 180502 (2021).

31. B. Villalonga, M. Y. Niu, L. Li, H. Neven, J. C. Platt, V. N. Smelyanskiy, S. Boixo, Efficient approximation of experimental Gaussian boson sampling. arXiv:2109.11525 [quant-ph] (23 September 2021).

32. J. J. Renema, A. Menssen, W. R. Clements, G. Triginer, W. S. Kolthammer, I. A. Walmsley, Efficient classical algorithm for boson sampling with partially distinguishable photons. *Phys. Rev. Lett.* **120**, 220502 (2018).

33. A. E. Moylett, R. García-Patrón, J. J. Renema, P. S. Turner, Classically simulating near-term partially-distinguishable and lossy boson sampling. *Quantum Sci. Technol.* **5**, 015001 (2020).

34. R. García-Patrón, J. J. Renema, V. Shchesnovich, Simulating boson sampling in lossy architectures. *Quantum* **3**, 169 (2019).

35. D. J. Brod, M. Oszmaniec, Classical simulation of linear optics subject to nonuniform losses. *Quantum* **4**, 267 (2020).

36. J. Shi, T. Byrnes, Gaussian boson sampling with partial distinguishability. arXiv:2105.09583 [quant-ph] (20 May 2021).

37. N. Quesada, L. G. Helt, J. Izaac, J. M. Arrazola, R. Shahrokhshahi, C. R. Myers, K. K. Sabapathy, Simulating realistic non-Gaussian state preparation. *Phys. Rev. A* **100**, 022341 (2019).

38. C. Weedbrook, S. Pirandola, R. García-Patrón, N. J. Cerf, T. C. Ralph, J. H. Shapiro, S. Lloyd, Gaussian quantum information. *Rev. Mod. Phys.* **84**, 621–669 (2012).

39. S. Olivares, Quantum optics in the phase space. *Eur. Phys. J. Spec. Top.* **203**, 3–24 (2012).

40. J. E. Gentle, *Computational Statistics* (Springer, 2009), vol. 308.

41. P. Clifford, R. Clifford, The classical complexity of boson sampling, in *Proceedings of the Twenty-Ninth Annual ACM-SIAM Symposium on Discrete Algorithms* (SIAM, 2018), pp. 146–155.

42. https://github.com/jakeffbulmer/gbs.

43. B. Gupt, J. Izaac, N. Quesada, The Walrus: A library for the calculation of hafnians, Hermite polynomials and Gaussian boson sampling. *J. Open Source Softw.* **4**, 1705 (2019).

44. S. K. Lam, A. Pitrou, S. Seibert, Numba: A llvmbased python jit compiler, in *Proceedings of the Second Workshop on the LLVM Compiler Infrastructure in HPC* (Association for Computing Machinery, 2015), pp. 1–6.

45. L. Dalcín, R. Paz, M. Storti, J. D'Elía, MPI for Python: Performance improvements and MPI-2 extensions. *J. Parallel Distrib. Comput.* **68**, 655–662 (2008).

46. L. Latmiral, N. Spagnolo, F. Sciarrino, Towards quantum supremacy with lossy scattershot boson sampling. *New J. Phys.* **18**, 113008 (2016).

47. P. Clifford, R. Clifford, Faster classical Boson Sampling. arXiv:2005.04214 [quant-ph] (7 May 2020).

48. M. Rudelson, A. Samorodnitsky, O. Zeitouni, Hafnians, perfect matchings and Gaussian matrices. *Ann. Probab.* **44**, 2858–2888 (2016).

49. D. S. Phillips, M. Walschaers, J. J. Renema, I. A. Walmsley, N. Treps, J. Sperling, Benchmarking of Gaussian boson sampling using two-point correlators. *Phys. Rev. A* **99**, 023836 (2019).
